# Homodimerization regulates an endothelial specific signature of the SOX18 transcription factor

**DOI:** 10.1093/nar/gky897

**Published:** 2018-10-18

**Authors:** Mehdi Moustaqil, Frank Fontaine, Jeroen Overman, Alex McCann, Timothy L Bailey, Paulina Rudolffi Soto, Akshay Bhumkar, Nichole Giles, Dominic J B Hunter, Yann Gambin, Mathias Francois, Emma Sierecki

**Affiliations:** 1 EMBL Australia node in Single Molecule Science and School of Medical Sciences, The University of New South Wales, Sydney, NSW 2031, Australia; 2 Institute for Molecular Bioscience, The University of Queensland, Brisbane, QLD 4072, Australia; 3 Department of Pharmacology, School of Medicine, University of Nevada, Reno, NV 89557, USA

## Abstract

During embryogenesis, vascular development relies on a handful of transcription factors that instruct cell fate in a distinct sub-population of the endothelium (1). The SOXF proteins that comprise SOX7, 17 and 18, are molecular switches modulating arterio-venous and lymphatic endothelial differentiation (2,3). Here, we show that, in the SOX-F family, only SOX18 has the ability to switch between a monomeric and a dimeric form. We characterized the SOX18 dimer in binding assays *in vitro*, and using a split-GFP reporter assay in a zebrafish model system *in vivo*. We show that SOX18 dimerization is driven by a novel motif located in the vicinity of the C-terminus of the DNA binding region. Insertion of this motif in a SOX7 monomer forced its assembly into a dimer. Genome-wide analysis of SOX18 binding locations on the chromatin revealed enrichment for a SOX dimer binding motif, correlating with genes with a strong endothelial signature. Using a SOX18 small molecule inhibitor that disrupts dimerization, we revealed that dimerization is important for transcription. Overall, we show that dimerization is a specific feature of SOX18 that enables the recruitment of key endothelial transcription factors, and refines the selectivity of the binding to discrete genomic locations assigned to endothelial specific genes.

## INTRODUCTION

Understanding how transcription factors (TFs) orchestrate gene expression to instruct a phenotypic output is fundamental to biology and future therapeutics. Dynamic control of gene transcription is particularly important during development as cell lineages are established. In mammals, many members of the SOX SRY-related High-Mobility Group (HMG) box family act as central regulators of gene expression to govern cell fate in a variety of key processes (4–7), such as vascular network assembly (8), cartilage formation and sex determination (9,10), neurogenesis (11), as well early stage development and embryonic stem cell pluripotency (12). These crucial roles are highlighted by the fact that many mutations in SOX genes cause severe congenital diseases in humans, such as XY sex reversal (SRY), campomelic dysplasia (SOX9), Waardenburg–Hirschsprung syndrome (SOX8) and anophthalmia–esophageal–genital syndrome (SOX2). A prominent feature of the SOX proteins is the presence of a 79 amino acids region which delineates the HMG-box, the DNA binding and bending domain. The HMG-box is present in all groups of SOX proteins (A-H, 20 SOX) and is classically used as a reference to align and compare these proteins since this region is highly conserved (7). It is made up of 3 α-helixes, whereby α1 and α2 are involved with DNA binding while α3 is involved in protein-protein interactions (13). The HMG-box recognizes a heptameric consensus sequence (5-A/TA/TCAAA/TG-3) on the DNA. The activity of SOX proteins at these binding locations is modulated by varying the combinations of protein-protein interactions which can cause activation or repression of transcription (14–16). In addition to the HMG-box common to all SOX genes, individual subgroups possess other functional domains that include: transactivation domain (TAD), coiled–coil, and proline-rich domains. The presence of these domains within the same group is likely to account for redundancy mechanism, an essential safety net to insure proper embryonic development (4). In particular, SOX proteins within the F group (SOX7, SOX17 and SOX18) regulate various aspects of vascular development (17–19) and often do so in a redundant manner (20).

Nevertheless, SOX18 is central to both angiogenesis and lymphangiogenesis (21). In human, several mutations in SOX18 are linked to the Hypotrichosis-Lymphedema-Telangiectasia and Renal Syndrome (HLTRS). HLTRS is a rare syndrome characterized by defects in hair follicle development (hypotrichosis), fluid accumulation in the limbs (lymphedema), presence of haemorrhagic blood vessels (telangiectasia), and renal defects as probands develop to adulthood. These features indicate that SOX18 function is required for the proper development of blood and lymphatic vasculature in human during embryogenesis (17,22). A series of *de novo* mutations causing HLTRS have been identified within the HMG domain and the TAD and have been associated to a broad spectrum of the syndrome severity (23). The murine counterpart of HLTRS is caused by natural mutations (allelic series: *Ragged* mice) in SOX18, which lead to truncated proteins. The truncated SOX acts as a dominant-negative protein that suppresses the endogenous activity of SOX7 and SOX17 (24,25). The phenotype of the *Ragged* mutant mice is characterized by severe vascular dysfunction, leading to the loss of endothelial cell junction integrity, which gives rise to a generalized haemorrhage, loss of lymphatic vascular function and a blockade of hair follicle cycle, mirroring the human syndrome (21). Despite an integral role for the SOX18 genetic pathway in vascular development there is a lack of information regarding its molecular mode of action.

Self-association, from dimers to higher-order oligomers, is often used by proteins to modulate activity and tune cellular responses (26). The capacity for self-association is even more significant for TFs since this ability modulates the physiological rate of gene transcription and may lead to deleterious effects when uncontrolled (3,27,28). It is particularly relevant in the case of SOX (29) proteins. An example of such a potent and functionally dynamic TF is SOX9. SOX9 can dimerize upon binding to DNA (30). Many studies have shown that the configuration of SOX9 as a monomer or a DNA-bound dimer leads to the binding of different enhancers, inducing differential gene transactivation (31,32). The relevance of the dimer function is dramatically illustrated in campomelic dysplasia disorder (33,34) where mutations interfere with SOX9 dimerization ability.

During a screen of small-molecules that could disrupt lymphangiogenesis in zebrafish, we showed that the SOX18 interaction network could be targeted pharmacologically (35). This work suggested that formation of SOX18 complexes is crucial for vascular development, and we set out to investigate the potential link between protein-protein interactions and target gene selectivity. In the present study, we used single molecule techniques and protein binding assays to study the behaviour of SOXF proteins *in vitro*. Here, we demonstrate that SOX18 has a unique ability to homodimerize, as opposed to other members of the F group. Using systematic truncation analysis, we identified and characterized a novel dimerization domain that is highly conserved during evolution. We validated this discovery *in vivo*, by developing a split-GFP biosensor of SOX18 dimerization in zebrafish larvae. Further, we found a specific palindromic doublet of SOX-binding consensus sequences in the human genome, evidence for the formation and importance of the SOX18 dimer. Gene ontology (GO) enrichment analysis of the subset of genes assigned to the SOX18 homodimer reveals a specific endothelial signature and include genes essential to vascular development. Finally, we validated that pharmacological disruption of SOX18 dimer interferes with the expression level of a subset of genes, linking physical interaction and transcriptional output.

### Materials and Methods

#### Plasmid preparation and cell free-expression

Proteins were genetically encoded with enhanced GFP (GFP), mCherry and cMyc (myc) tags, and cloned into the following cell free expression Gateway destination vectors: N-terminal GFP tagged (pCellFree_G03), N-terminal Cherry-cMyc (pCellFree_G07) and C-terminal Cherry-cMyc tagged (pCellFree_G08) (36). Human RBPJ (Q06330 SUH_HUMAN), GATA 2 (P23769) and MEF2C (BC026341) Open Reading Frames (ORFs) were sourced from the Human ORFeome collection, versions 1.1 and 5.1, and the Human Orfeome Collaboration OCAA collection (Open Biosystems), as previously described (37) and cloned at UNSW. The entry clones pDONOR223 vectors were exchanged with the ccdB gene in the expression plasmid by LR recombination (Life Technologies, Australia). The full-length human *SOX18* gene was synthesized (IDT DNA, USA) and transferred to pCellFree vectors using Gateway cloning. Translation competent *Leishmania tarentolae* extract (LTE) was prepared as previously described (38,39). GFP- and Cherry-tagged proteins were expressed separately for 15 min at 27°C to start transcription, then were mixed and co-expressed for 3 h.

#### Preparation of the SOX18 truncation constructs

The DNA sequences encoding the desired domains were obtained by PCR amplification of the SOX18 WT construct with the combination of primers listed in Supplementary Table S1. PCR amplification was performed using Phusion polymerase. The PCR fragments were isolated by electrophoresis and purified using Promega Wizard^®^ SV gel and PCR clean up system. These fragments were then cloned into the Gateway destination vectors (pCellFree_G03 or pCellfree_G08) by LR recombination (Life Technologies, Australia) as described previously.

#### Construction of the SOX18DIM/SOX7 swap construct

The SOX18DIM/SOX7 swap construct was made by exchanging the 50 amino acids following the HMG box of SOX18 WT:

Y R P R R K K Q A R K A R R L E P G L L L P G L A P P Q P P P E P F P A A S G S A R A F R E L P P L

with the 50 amino acids following the HMG box of SOX 7 WT

Y R P R R K K Q A K R L C K R V D P G F L L S S L S R D Q N A L P E K R S G S R G A L G E K E D R G

The swap construct was obtained as a gBlock (IDT), and was exchanged with the ccdB gene in the donor plasmid (pDONOR223) by BP recombination (Life Technologies, Australia), then with the ccdB gene in the expression plasmid (pCellFree_G03 and pCellFree_G08) by LR recombination (Life Technologies, Australia) as described previously.

#### Construction of the SOX7DIM/SOX18 swap construct

The SOX7DIM/SOX18 swap construct was created by exchanging the 50 amino acids following the HMG box of SOX7 WT:

Y R P R R K K Q A K R L C K R V D P G F L L S S L S R D Q N A L P E K R S G S R G A L G E K E D R G

with the 50 amino acids following the HMG box of SOX 18WT

Y R P R R K K Q A R K A R R L E P G L L L P G L A P P Q P P P E P F P A A S G S A R A F R E L P P L

The swap construct was obtained as a gBlock (IDT), and was exchanged with the ccdB gene in the donor plasmid (pDONOR223) by BP recombination (Life Technologies, Australia), then with the ccdB gene in the expression plasmid (pCellFree_G03 and pCellFree_G08) by LR recombination (Life Technologies, Australia) as described previously.

#### Multiple sequence alignment

Putative SOX18 homodimeriation domains from 9 different species (*Mus musculus, Danio rerio, Xenopus tropicalis, Gallus gallus, Anolis carolinensis, Orcinus orca, Monodelphis domestica, Latimerica chalumnae* and *Callorhinchus milii*) were obtained using the 50 amino acid human SOX18 homodimerization domain as a query in Protein Blast (NCBI). Multiple sequence alignment of the SOX18 homodimer domain of 10 different species (including human), as well as the corresponding 50 amino acid region of the human SOXF family (SOX7, SOX17 and SOX18) was performed using Clustal Omega.

#### AlphaScreen assay

The assay was performed as previously described (37,40), using the cMyc detection kit and Proxiplate-384 Plus plates (PerkinElmer). The LTE lysate co-expressing the proteins of interest was diluted in buffer A (25 mM HEPES, 50 mM NaCl). For the assay, 12.5 μl (0.4 μg) of Anti-cMyc coated Acceptor Beads in buffer B (25 mM HEPES, 50 mM NaCl, 0.001% NP40, 0.001% casein) were aliquoted into each well. This was followed by the addition of 2 μl of diluted sample, at different concentration, and 2 μl of biotin labeled GFP-Nanotrap in buffer A. Then 2 μl (0.4 μg) of Streptavidin coated Donor Beads diluted in buffer A was added, and the plate was incubated in the dark for 1.5h min at room temperature. The AlphaScreen signal was measured on an Envision Plate Reader (PerkinElmer), using manufacturer's recommended settings (λ_exc_ = 680/30 nm for 0.18 s, λ_em_ = 570/100 nm after 37 ms). The resulting bell-shaped curve is an indication of a positive interaction, while a flat line reflects a lack of interaction between the proteins. Measurements of each protein pair were performed in triplicate. A binding index was calculated as: ${\rm BI}\ = \left[ {\frac{{I - {I_{{\rm neg}}}}}{{{I_{{\rm ref}}} - {I_{{\rm neg}}}}}} \right]{\rm{\ }} \times {\rm{\ }}100$, where *I* is the highest signal level (top of the hook effect curve) and *I*_neg_ is the lowest (background) signal level. The signals were normalized to the *I*_ref_ signal obtained for GFP-SOX18/SOX18-Cherry-myc.

All experiments were performed using independent and technical triplicates (*N* = 6, *n* = 3).

#### Single-molecule spectroscopy

GFP- and Cherry-tagged SOX18 proteins were expressed separately for 15 min at 27°C to initiate the transcription and then were mixed and co-expressed for 3 h. 20 μl samples were used for each experiment. These were placed into a custom-made silicone 192-wells plate equipped with a 70 × 80 mm glass coverslip (ProSciTech Australia). Plates were analysed on a Zeiss LSM710 microscope with a Confocor3 module, at room temperature. Two lasers (488 and 561 nm) were co-focussed in the well solution using a 40 × 1.2 NA water immersion objective (Zeiss, Germany); fluorescence was collected and split into GFP- and Cherry-channel by a 560 nm dichroic mirror. The GFP emission was further filtered by a 505–540 nm band pass filter and the Cherry emission was filtered by a 580 nm long-pass filter (41).

#### Plasmid preparation of BiFC reporters for *in vivo* expression

mVENUS-based FosLZ/JunLZ plasmids (pCS2+-NmVENUS155-FosLZ and pCS2+-CmVENUS155-JunLZ, courtesy of Dr Andrew Badrock) were used as a starting point to construct the SOX18 homodimer BiFC reporters (NmVENUS155-SOX18S and CmVENUS155-SOX18S) and the SOX18 homodimer mutant BiFC reporters used as a negative control (NmVENUS155-SOX18SΔ84-205 and CmVENUS155-SOX18SΔ84-205). pCS2+-NmVENUS155-SOX18S and pCS2+-CmVENUS155-SOX18S were generated using In-Fusion cloning (In-Fusion HD EcoDry Cloning Kit, Clonetec) by replacing FosLZ and JunLZ with SOX18S. Circular polymerase extension cloning (CPEC) was used to remove the HMG, NLS and homodimerization domains of SOX18S (SOX18SΔ84–205), which was then inserted into the pCS2+-mVENUS constructs via In-Fusion cloning to generate pCS2+-NmVENUS155-SOX18SΔ84-205 and pCS2+-CmVENUS155-SOX18SΔ84-205.

#### *in vitro* mRNA synthesis of BiFC reporters and microinjection into zebrafish embryos

Restriction enzyme digestion was performed to linearize 5 μg of each mVENUS-based BiFC reporter construct. Following linearization, BiFC reporter DNA was purified (DNA Clean & Concentrator™-5 Kit, Zymo), 1 μg of which was used as a template for *in vitro* mRNA synthesis (mMESSAGE mMACHINE SP6 RNA Synthesis Kit, Ambion). Synthesized BiFC reporter mRNA was purified (RNA Clean & Concentrator™-5 Kit, Zymo) and 1 nl of 100 ng/μl mRNA was co-injected with phenol red into the yolk sac of single-cell zebrafish embyros. Embryos were maintained in E3 media (5.0 mM NaCl, 0.17 mM KCl, 0.33 mM CaCl, 0.33 nM MgSO_4_) at 28°C until they reached 4–5 hpf.

#### Zebrafish embryo imaging

4–5 hpf zebrafish embryos were screened for fluorescence using a fluorescent stereo microscope (M165FC, Leica). Embryos identified as fluorescent had the chorion manually removed prior to being embedded in a 2% methylcellulose-containing 35 mm glass-bottom dish. Zebrafish embryos were imaged live using confocal laser scanning microscopy (LSM710, Zeiss), whereby a 514 nm laser was used to visualise mVENUS-based BiFC reporters. Fluorescent and brightfield images were taken as Z-stacks at 10 × magnification with a 0.45 NA dry objective and at 40 × magnification with a 1.3 NA oil objective. Post-acquisition image processing was performed using FIJI (FIJI Is Just ImageJ) to generate maximum intensity projections and fluorescence/brightfield composites. Time-lapse images were taken over a 10 h period.

#### Purified full-length mouse SOX18

A cDNA clone of mouse *Sox18* was PCR amplified and cloned into the pOPIN-GST vector, to generate N-terminally tagged HIS-GST-SOX18. A sequence-verified construct was co-transfected with *flash*BACULTRA (Oxford Expression Technologies, Oxford, United Kingdom) baculovirus DNA onto *Spodoptera frugiperda* Sf9 cells to obtain recombinantly expressed HIS-GST-SOX18. High Five cells (BTI-TN-5B1-4) in Sf-900™ II serum-free medium were infected at cell density of 1.5 × 10^6^ cells/ml with a multiplicity of infection (MOI) of 5 PFU/cell, and incubated at 21°C for 144 h before harvest. The cell pellet from 100 mL of expression culture was resuspended in 30 mL of phosphate lysis buffer (50 mM sodium phosphate, 500 mM sodium chloride, 1% Triton X-100, 2 mM magnesium chloride, one tablet of cOmplete Protease Inhibitor Cocktail, pH 7.5) and sonicated on ice for 20 s. The cell lysate was centrifuged at 17 000 × *g* for 40 min at 4°C. Supernatant was incubated with Benzonase Nuclease (Merck Millipore) for 1 h at room temperature for DNA degradation, before being mixed with 500 μl GST resin (GE Healthcare Life Sciences, Sweden) and incubated on a rotating wheel at room temperature for 1 h. The sample was centrifuged at 500 × *g* for 1 min to remove unbound protein in the supernatant. The resin was further washed with 50 resin volumes (RV) wash buffer (50 mM sodium phosphate, 500 mM NaCl, pH 7.5), with unbound protein removed by centrifugation as above. Bound protein was eluted from the resin with 3 × 1 RV elution buffer (50 mM Tris, 500 mM NaCl, 10 mM reduced glutathione, pH 8.0), collecting the supernatant by centrifugation as above.

#### Purified mouse SOX HMG fragments

The HMG domain of mouse SOX18 was BP cloned from cDNA templates (IMAGE cDNA clone IDs: *Sox18*: 3967084) into a pDONR™221 pENTRY vector, sequenced and recombined into a pETG20A or a pHisMBP expression plasmid using Gateway®LR Technology (42). Constructs were transformed into *Escherichia coli* BL21(DE3) cells (Luria-Bertani, 100 μg/ml Ampicillin) and purified as described above.

#### Design of the synthetic palindromic probes

A double-strand (ds) 37 bp-long DNA probe centred on a synthetic IR5 element was designed. GC-rich flanking and spacer sequences were used to avoid confounding off-site protein–DNA binding. Three spacer lengths were designed: 1 (IR1), 5 (IR5) and 10 (IR10) bp. The DNA probes were obtained from IDT (IDT DNA, USA).

Sequences for the probes are:

**Table:** 

IR10:	+ve	cgccagtAACAATagggcggcttATTGTTccgggggc-
	−ve	gcggtcaTTGTTAtcccgccgaaTAACAAggcccccg-
IR5:	+ve	cgccagtaggAACAATgcggcATTGTTttccgggggc-
	−ve	gcggtcatccTTGTTAcgccgTAACAAaaggcccccg-
IR1:	+ve	cgccagtagggcAACAATgATTGTTgcttccgggggc-
	−ve	gcggtcatcccgTTGTTAcTAACAAcgaaggcccccg-

#### EMSA (electrophoresis mobility shift assay)

EMSAs were performed using a DNA elements with 5′ cy5 (Cyanine 5) label (Sigma Proligo) and Sox18_79, a protein construct encoding 79 amino acids of the HMG-domain of mouse Sox18. Experiments were carried out by incubating 15 nM mSox18 HMG with 1 nM DNA in binding buffer (20 mM Tris–HCl pH 8.0, 50 μM ZnCl_2_, 100 mM KCl, 10% glycerol, 2 mM β mercaptoethanol, 0.1 mg/ml bovine serum albumin (BSA), 0.1% (v/v) NP-40 and 5% DMSO) in a reaction volume of 10 μl, for 1 h at 4°C in dark. Samples were loaded into a pre-run 12% (w/v) 1× Tris-glycine polyacrylamide gel, electrophoresed in 1× TG (25 mM Tris, pH 8.3; 192 mM glycine) buffer at 150 V for 50 min at 4°C and visualized by phosphorimaging (Typhoon 9410, Amersham Bioscience).

#### Fluorescence polarization assay

Protein-DNA binding was measured by fluorescence polarization, using fluorescein 5′-phosphate-tagged ds DNA probes. Three spacer lengths were tested: 1, 5 and 10 bp. The DNA-binding assay was performed in 20 μl, in black 384-well plates, using mouse full-length SOX18, or a SOX-HMG fragment incubated in 30 mM HEPES buffer pH 7.5, supplemented with 100 mM KCl, 40 mM NaCl, 10 mM ammonium acetate, 10 mM guanidinium HCl, 2 mM MgCl_2_, 0.5 mM EDTA, and 0.01% Nonidet NP-40. Protein concentrations ranging from 5 to 150 nM, in presence of a constant 5 nM labelled DNA. Controls consisted of: free labelled DNA (low FP milli-Polarization index, mP); labelled DNA in presence of protein (negative control, high mP); labelled DNA and protein in presence of 400 times excess of unlabelled DNA (positive control, low mP). Plates were sealed, briskly agitated in the dark at room temperature for 5 min then centrifuged at 1800 rpm for 10–20 s to flatten the sample meniscuses. Plates were allowed to equilibrate for another 15 min at room temperature, before reading fluorescence polarization on a Tecan M1000Pro (λ_exc_ = 485 nm, λ_em_ = 525 nm). All experiments were performed using independent and technical triplicates (*N* = 3, *n* = 3).

At given constant temperature and viscosity, the fluorescence polarization index (mP) is proportional to the molecular size of binding complex. Binding data were fitted to the Hill equation using GraphPad Prism version 7.03 for Windows, GraphPad Software (La Jolla, CA, USA).

#### Effect of the palindromic sequence or Sm4 on protein-protein interaction

Disruption of protein-protein interactions was assayed to obtain IC50 values by expressing the desired protein pairs in LTE and incubating with IR5 or with the small molecule **Sm4**, dissolved in DMSO, at different final concentration. Control incubations used 0.7% (v/v) DMSO final concentration for **Sm4**. Incubations were in buffer B for 1 h. Percentage of interaction was calculated as: $ \left( {\frac{{{I_{{\rm cpd}}}}}{{{I_{{\rm DMSO}}}}}} \right) \times \ 100.$ Data from three independent experiments were fitted in GraphPad Prism version 6.0 using three-parameter non-linear regression.

#### SpaMo analysis

SpaMo analysis was performed on ChIP-seq peaks using a UniPROBE SOX18 motif (UP00064_1, consensus sequence 5′-AATTGTTNT-3′ as the ‘primary’ motif, and the complete set of UniPROBE motifs as the ‘secondary’ motif set. The input to SpaMo was repeat-masked 500 bp regions centered on each of the 23 635 SOX18 ChIP-seq peaks. The exact SpaMo command used was:

spamo -oc results/jc2454_HUVEC_myc_SOX18_merge_hg19_homer.500bp.minscore.5.margin.150.range.20.trim.1-keepprimary.UP00064_1.Sox18_primary.bg.input.spamo -numgen 1 -keepprimary -minscore 5 -margin 150 -range 20 -trim 1 -bgfile /Users/t.bailey/Genomes_local/hg19/Homo_sapiens.GRCh37.66.dna.toplevel.bg tmp/jc2454_HUVEC_myc_SOX18_merge_hg19_homer.500bp.fa data/motifs/UP00064_1.Sox18_primary.meme

#### RT-PCR Dose effect of Sm4 treatment

Total RNA was extracted using a RNeasy Mini kit (Qiagen, 74106) according to the manufacturers protocol, including on column DNA digestion. cDNA was synthetised from 1μg of purified RNA using the High Capacity cDNA Reverse Transcription kit (Life Technologies, 4368813). Amplification and quantitation of target cDNA was performed in technical triplicates of at least three biological replicates using the SYBR green (Life Technologies, 4312704) method. Reactions were run in 10 μl in 384-well plates using a ViiA 7 Real-Time PCR system. The housekeeper gene *GAPDH* was selected based on the stability of their expression after validation by cross-referencing against expression of other housekeeper genes including 18s ribosomal RNA and beta-actin. Primer efficiencies were calculated using LinRegPCR, and amplification data was analysed using ViiA7 software and the Q-gene PCR analysis template.

## RESULTS

### Formation of complexes within the SOXF group

To assess whether members of the SOXF group have the potential to self-interact, we first measured physical interactions using different *in vitro* assays. Transcription factors are notoriously difficult proteins to work with, and SOXF proteins are no exception. The small DNA-binding domain can be expressed and purified in recombinant form, but the full-length proteins are difficult to obtain. The N-terminal and C-terminal domains of SOX18 are likely intrinsically disordered, reducing further the probability of high-resolution structural studies using crystallography.

Therefore, in order to characterize the behaviour of full-length SOX7, SOX17 and SOX18 proteins, we turned to cell-free protein translation. In recent years, our laboratory has successfully expressed and studied difficult targets using a eukaryotic cell-free system based on *Leishmania tarentolae* (39,40,43). When supplemented with plasmids encoding the SOXF proteins, the cell-free system produces full-length proteins in 3 hours, with minimal truncations (Supplementary Figure S1).

One of the advantages of cell-free expression is the ability to co-express different constructs, and we used this to investigate protein self-oligomerisation. Specifically, we co-expressed GFP- and mCherry-tagged SOXF proteins and used the two tags for affinity capture and single-molecule fluorescence detection. The proteins were labelled at either end (N- or C-terminal) to assess the effect of the fluorophore on protein-protein interactions (PPIs) (Supplementary Figure S2).

**Figure 1.**
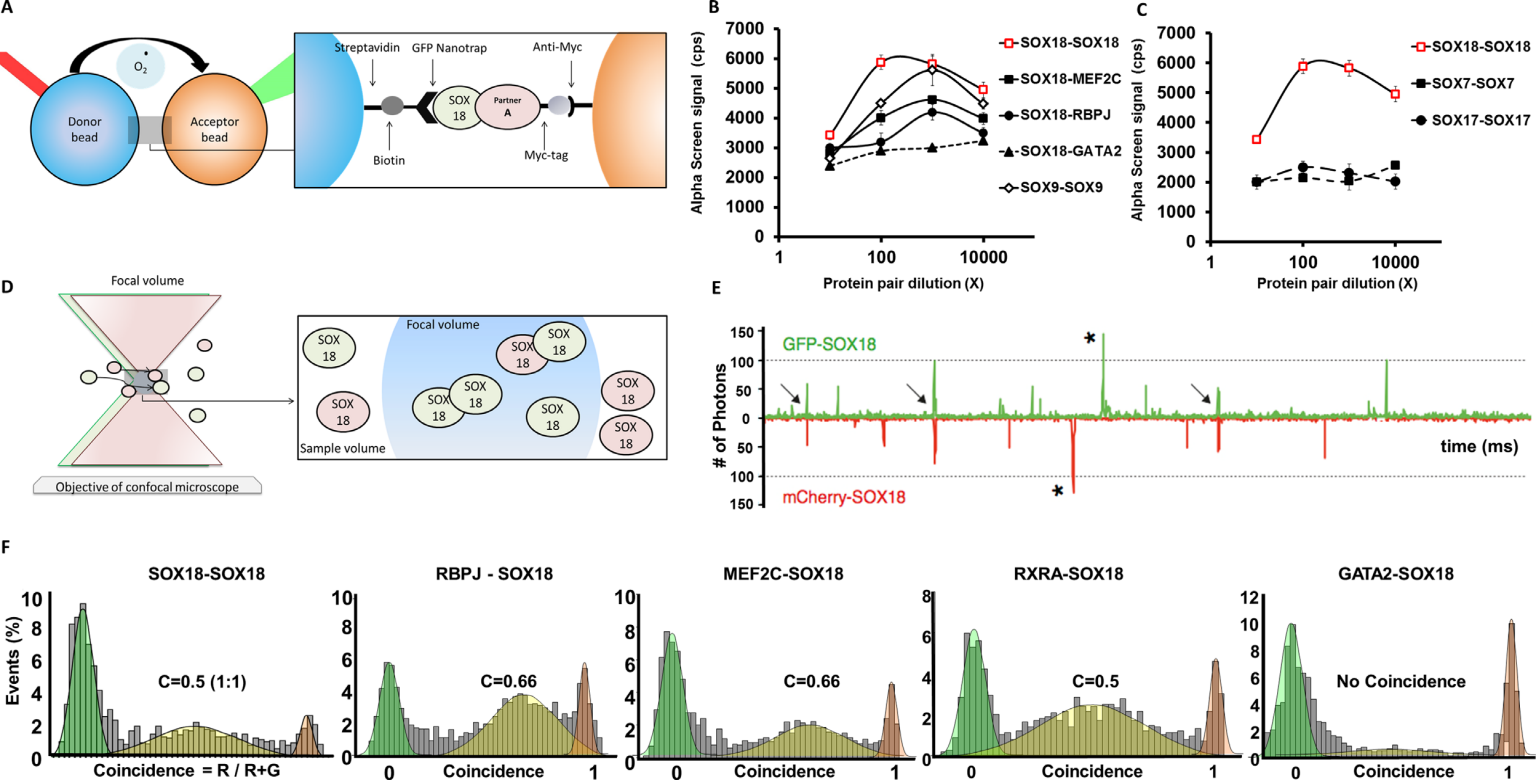
In Vitro characterization of the SOX18 dimer. (**A**) Schematic representation of the AlphaScreen assay showing interaction between SOX18 and co-factor A. This bead–bead assay relies on transfer of singlet oxygen from a donor bead to a luminescent acceptor bead when protein–protein interactions bring the beads within 200 nm (37) (see ‘Materials and methods’) (**B**) Typical AlphaScreen curves obtained for a non-interacting pair (GFP-SOX18 and GATA2-Cherry-myc) and for interacting pairs such the homodimer of SOX9 and GFP-SOX18 with respectively mCherry-myc SOX18, MEF2C and RBPJ. (**C**) Representative plots of AlphaScreen signal as a function of concentration for SOX18, SOX7 and SOX17 homodimers. (**D**) Schematic representation of single-molecule fluorescence experiment in which the proteins freely diffuse in and out of the focal volume created by two lasers simultaneously exciting the GFP and Cherry fluorophores. (**E**) Example of a single-molecule trace obtained after co-expression of SOX18-GFP and SOX18-Cherry. The numbers of photons detected in green and red channels are plotted as a function of time. The trace shows simultaneous bursts in both GFP and Cherry channels that reflect formation of dimers containing both fluorophores (arrows). (**F**) Histogram of single-molecule coincidence between respectively SOX18-GFP and SOX18-Cherry, MEF2C-Cherry, RBPJ-Cherry, RXRA-Cherry and GATA2-Cherry. In this experiment, GFP-labelled and Cherry-labelled protein were expressed separately in LTE then mixed together and allowed to interact for 1h before the assay. In all cases, the mixtures were diluted to pM concentrations immediately before testing. A fluorescence signal was recorded in the GFP channel and the Cherry channel over 500 s. The signal was then analyzed as a succession of individual events. For each event, a ratio of Cherry fluorescence to the total fluorescence is calculated. The number of events for each ratio C was counted and normalized to the total number of events. This fraction of events P(C) is plotted as a function of coincidence ratio (C). Gaussian curves are overlayed on the histograms: the green Gaussian curve corresponds to GFP only, the red Gaussian to Cherry only; the yellow Gaussian highlights the presence of both GFP and Cherry in the focal volume.

First, we performed a proximity assay (AlphaScreen, AS) to measure interaction between protein pairs. The assays were performed directly from the cell-free co-expressions, without enrichment or purification steps that could perturb weak complexes. In AS, the interaction between the two proteins brings donor and acceptor beads into close proximity, generating a luminescent signal (Figure 1A). The amplitude of the signal produced is proportional to the degree of physical interaction between two proteins. Previously reported interactions such as the SOX9 dimer (44), SOX18-MEF2C (45) and SOX18-RBPJ (35) were used as positive controls (Figure 1B) whereas the known lack of interaction between SOX18 and GATA2 was used to define a baseline level for the AS signal. When testing the SOXF group, AS revealed a strong binding between the SOX18-GFP/SOX18-mCherry pair while SOX7 and SOX17 did not form homodimer complexes (Figure 1C). To verify that the genetically encoded tags did not prevent interaction, we tested different configurations of the fluorophores in this assay and identified that the N-GFP-SOX18/ SOX18-C-mCherry pair gave the strongest AS signal. For SOX18, all other configurations did lead to a positive, albeit weaker AS signal, while none of the combinations in the case of SOX7 and SOX17 yielded a positive AS signal (Supplementary Figure S2).

To further characterise SOX18 dimer complexes and their ability to recruit protein partners, we took advantage of single molecule spectroscopic assays. This approach measures the photon emission of individual molecules of GFP or mCherry in a defined confocal volume (Figure 1D). After co-expression of GFP and mCherry tagged SOX18 proteins, the samples were rapidly diluted to working concentrations of approximately 100 pM. In these conditions, individual protein complexes are observed as they travel through the confocal excitation volume. A single GFP or mCherry fluorophore can emit a maximum of 90–100 photons per millisecond (40), and we used this calibration to quantify the size of complexes. In the trace obtained for GFP and mCherry tagged SOX18, we did not detect large bursts of fluorescence (>200 photons) that would indicate that the proteins form large oligomers or non-specific aggregates. We did observe the presence of slightly larger bursts in both GFP and mCherry channels, with intensities in the 100–200 photon range (Figure 1E, arrows). These bursts suggest the formation of SOX18 complexes containing two GFP or two mCherry fluorophores.

This observation was further confirmed using two-colours coincidence detection, as shown in Figure 1E. The fluorescence trace shows frequent co-diffusion of two SOX18 proteins tagged separately with GFP and mCherry. At these concentrations, the random simultaneous presence of two proteins in the small detection volume is highly improbable. Thus, the method provides a direct visualization of protein-protein binding. In the single molecule coincidence assay, binding stoichiometry can be inferred by measurement of the coincidence ratios of the protein complexes. By simply measuring the fraction of mCherry in the total fluorescent bursts, protein stoichiometries can be plotted, which clearly show that SOX18 forms a 1:1 dimer with a coincidence ratio *C* = mCherry/(GFP + mCherry) = 0.5 (Figure 1F).

Taken together, AS and single molecule coincidence results firmly demonstrate that SOX18 has the ability to form a dimer, unlike SOX7 or SOX17.

### SOX18 dimer recruits specific protein partners

The identification of SOX18 homodimers prompted us to determine the stoichiometric ratios for different assembly complexes formed with protein partners such as MEF2C, RBPJ and RXRA (Figure 1F). In this assay, we used GATA2 as a negative control for SOX18 interaction. The frequency distribution of coincidence ratio between mCherry-SOX18 and GFP-tagged MEF2C or RBPJ correspond to 2:1 interaction (*C* = 0.66), whereas binding to RXRA occurs in a 1:1 ratio (*C* = 0.5). These data provide evidence that the SOX18 dimers recruit MEF2C or RBPJ whereas monomeric SOX18 is able to recruit RXRA monomers.

### SOX18 homodimer forms *in vivo* in zebrafish larvae

**Figure 2.**
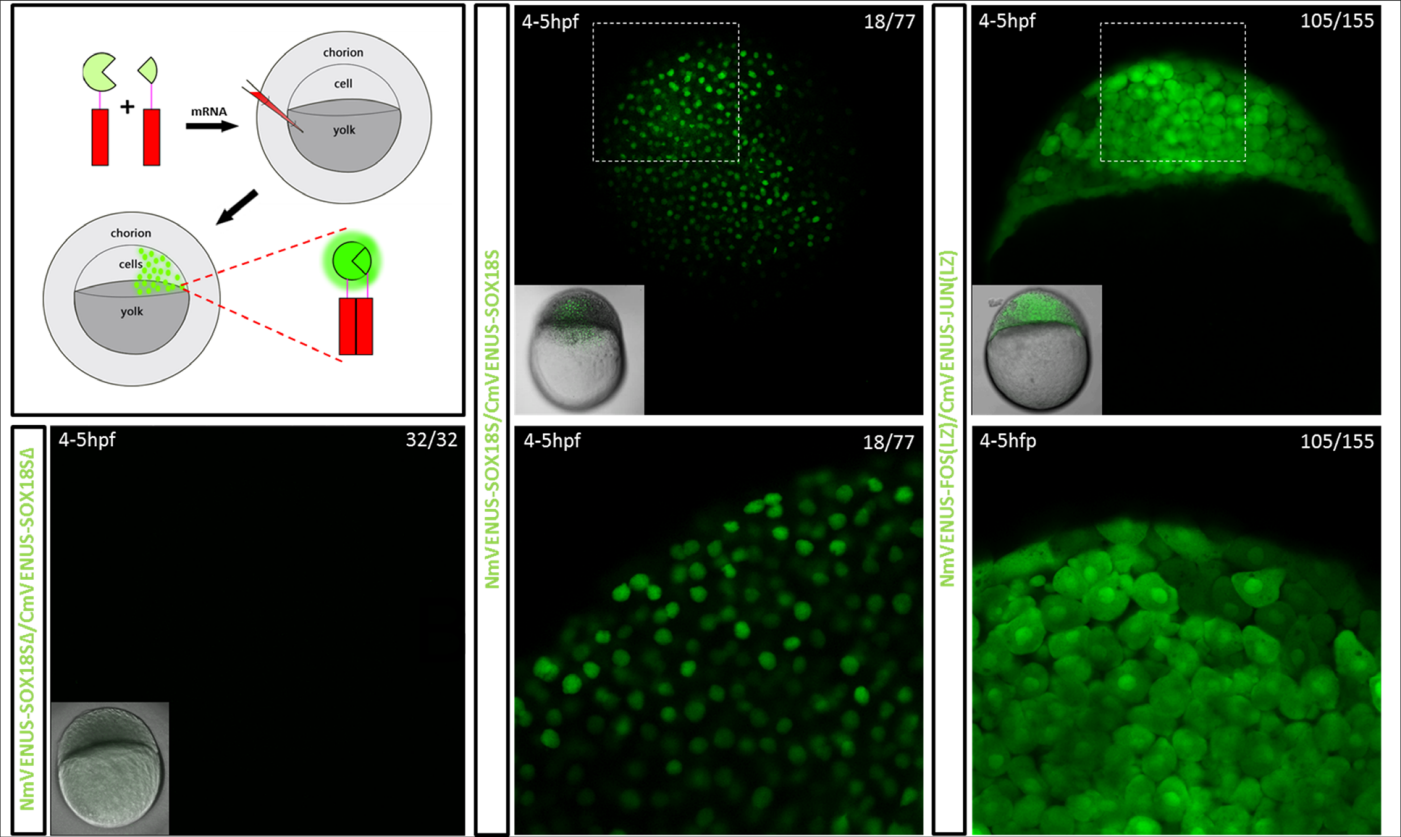
Formation of the SOX18 homodimer during embryogenesis in a zebrafish mode using a split GFP reporter. **Top left panel, s**chematic representation of the experiment. mRNAs encoding the two parts of the BiFC reporter were injected into 1 cell stage zebrafish larvae and imaged live via confocal scanning laser microscopy. **Bottom left panel**, negative control. Constructs of SOX18 that lack the NLS and HMG domains (Δ84–205) do not dimerize and no fluorescence is detected. **Middle panel**, visualisation of SOX18 homodimers. **Right panel**, JUN/FOS leucine zipper (LZ) domain heterodimers, in 4–5hpf developing zebrafish embryos. SOX18 homodimer split-FP biosensor fluorescence is only observed within cell nuclei, whereas FOS/JUN LZ heterodimer biosensor fluorescence is observed in both the cytoplasm and nuclei.

As a demonstration that SOX18 has the capability to homodimerise *in vivo* during development, we investigated the dimer formation using a zebrafish-based model system. To follow the formation of the SOX18 dimer in developing zebrafish larvae, we engineered a fluorescent reporter based on a split-fluorescent protein (split-FP) biosensor and took advantage of this construct in transient transgenic reporter experiments. Bimolecular fluorescence complementation (BiFC) assays are powerful tools for the visualisation of protein-protein interactions in both cell and zebrafish model systems (46,47). We found that a mVENUS-based split-FP biosensor was the most suitable for use in zebrafish embryos for the visualisation of SOX18 dimerization events. The selected mVENUS biosensor incorporates the N-terminus of mVENUS fragmented at amino acid 155 on the N-terminus of one SOX18 (NmVENUS155-SOX18), and the C-terminus of mVENUS fragmented at amino acid 155 on the N-terminus of another SOX18 (CmVENUS155-SOX18). These fragments were tagged to SOX18 via a flexible 3xGGGS linker (Supplementary Figure S3). The mRNA encoding these biosensors was co-injected into zebrafish embryos at the single-cell stage to promote ubiquitous expression of this TF during early stage development (Figure 2, top left panel).

Live imaging of the biosensor-injected larvae at around 4–5 hpf revealed the presence of mVENUS expression specifically in the nuclei (Figure 2, middle panel and Movie 1). In parallel, FosLZ/JunLZ heterodimers coupled to the BiFC reporter system were used as a positive control. Zebrafish embryos injected with a similar concentration of this FOSLZ/JunLZ biosensor mRNA display fluorescence in both nuclear and cytoplasmic localisations at the same developmental stage (Figure 2 right panel). To further validate the specificity of the split-FP biosensor assay, we established a negative control using a truncated version of SOX18 protein that does not harbour the HMG-box and nuclear localisation sequence (NLS) (Δ84-205). Transgenic zebrafish embryos expressing this mutant split-FP biosensor did not show any fluorescent signal in cell nuclei (Figure 2 bottom left panel). Therefore, the use of a BiFC reporter system further confirmed the capability of SOX18 to form a dimer *in vivo*.

### Mapping of Sox18 dimerization domain

**Figure 3.**
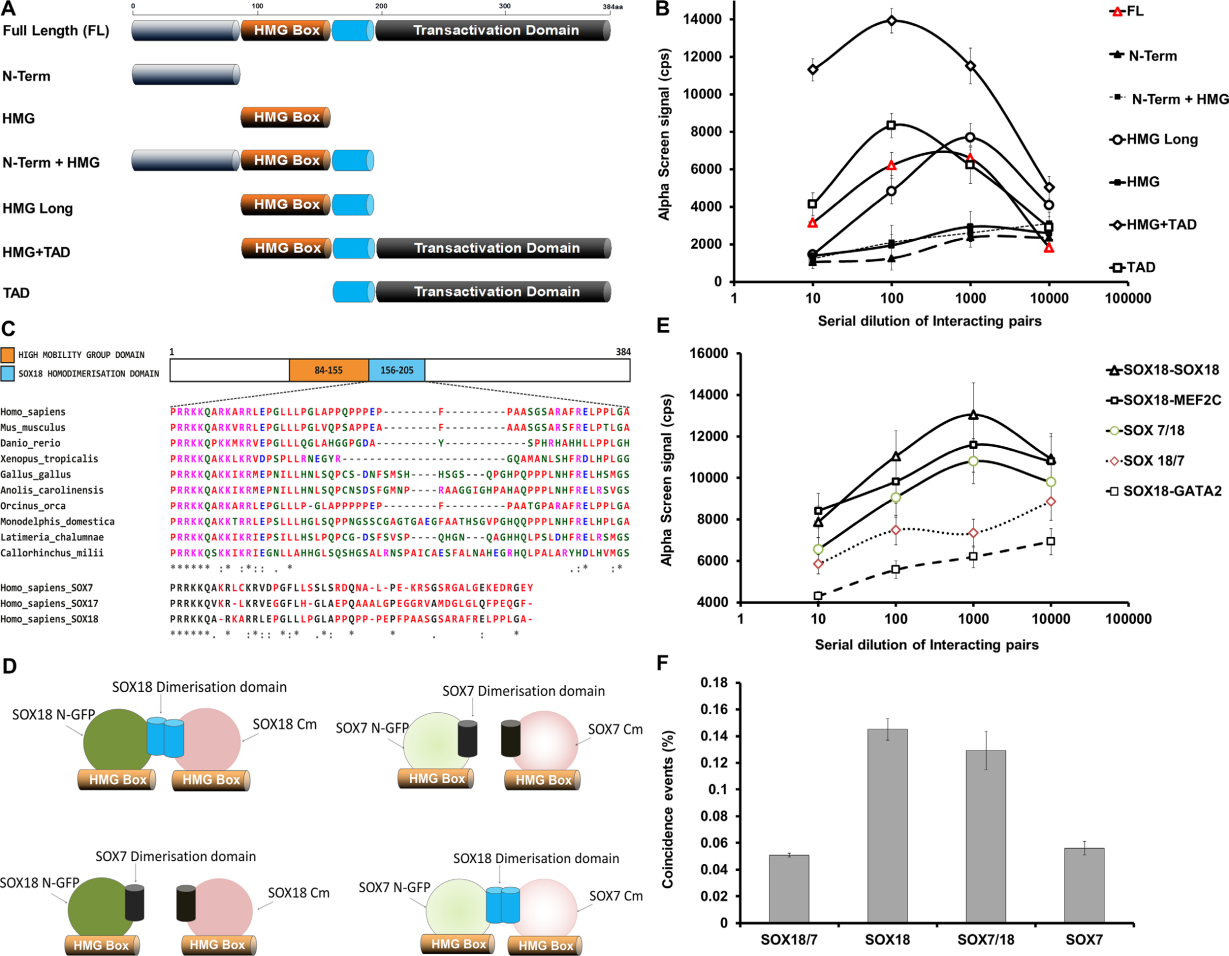
Biochemical characterization of the dimerization domain of SOX18. (**A**) Schematic representation of 7 mutants representing domain specific truncations of SOX18. (**B**) Typical curves of AlphaScreen signal as a function of concentration for the seven SOX18 truncations. Only FL, HMG-TAD, HMG long and TAD form dimer whereas N-Term, N-Term-HMG and HMG show no signal. (**C**) **Top**: multiple sequence alignment of the SOX18 homodimerization domain across 10 different species, using the human SOX18 homodimer domain as a reference. Residues are grouped into colours, based on their chemical and physical properties. **Bottom:** multiple sequence alignment of the putative SOX18 dimerization domain with the corresponding domains in SOX17 and SOX7 proteins. The 50 amino acids directly following the high mobility group (HMG) domain of the SOXF protein family reveals residues that are non-conserved and therefore possibly involved in the unique ability for SOX18 to homodimerize. Residues that are not conserved between all SOXF family members are highlighted in red. For all, fully conserved residues are marked by an asterisk (*), partially conserved residues that retain high similarity are marked by a colon (:), partially conserved but weakly similar residues are marked by a full stop (.) and residues that have no conservation are left blank (). Protein alignment was performed using Clustal Omega. (**D**) Schematic representation of the constructs: (top) SOX18 WT and SOX7 WT; (bottom) SOX18DIM/SOX7 and SOX7DIM/SOX18 swaps. (**E**) Typical AlphaScreen curves obtained for SOX18 WT with MEF2C and SOX7DIM/SOX18 showing respectively a positive signal above 10 000 cps. Lack of signal for the SOX18DIM/SOX7 swap indicates a loss of the dimerization propensity. SOX18-GATA2 is used as a negative control. (**F**) Value of coincidence obtained from the two-colours coincidence experiments performed on SOX18 WT, SOX18/7, SOX7/18 and SOX7 WT co-expression as a GFP/Cherry pair. Data were analysed as in Figure 1 and the percentage of coincident events (0.25 < *C* < 0.75) was plotted for the different constructs, reflecting their ability to homodimerize.

To pinpoint a putative SOX18 dimerization (DIM) domain, we generated a series of truncated constructs and tested their binding ability in AS. The truncations were designed based on the known domains of SOX18 full-length, as shown in Figure 3A. Truncated constructs included: [N-terminus], [HMG box], [N-terminus + HMG box], [HMG box long], [HMG box + TAD] and [TAD]. As shown in Figure 3B, AS revealed that SOX18 dimerization only occurred in the presence of a region corresponding to a 50 amino acids sequence (aa 156 to 205) predicted to link the HMG-box and the transactivation domain. The level of conservation of the DIM domain across species is shown in Figure 3C. This 50 amino acids region is highly conserved throughout evolution in SOX18. However, this region is not conserved in SOX7 or SOX17.

To validate the importance of this region in the dimerization process (Figure 3D), we swapped the 50 amino acids post-HMG-box of SOX18 with the corresponding 50 amino acids of SOX7 (SOX18DIM/SOX7-swap mutant). We also performed the reciprocal experiment whereby the putative SOX18 DIM domain was inserted into the corresponding site on the SOX7 protein. This chimeric protein corresponds to a SOX7DIM/SOX18-swap mutant. The SOX7 sequence was used since this TF was shown not to dimerize in AS and single molecule spectroscopy assays.

The homodimerization ability of the two swap mutants were tested in AS (Figure 3E) and single molecule two-colours coincidence (Figure 3F). In both assays, insertion of the exogenous SOX7 region into SOX18 caused a loss of interaction, indicating that this 50 amino acids region encompasses a motif that is necessary to the dimerization process. Conversely, insertion of the SOX18 DIM domain enabled the SOX7DIM/SOX18 swap mutant to homodimerize when SOX7 WT does not. These experiments establish that the DIM domain is sufficient to drive the dimerization process. The fact that dimerization is not restored to the same level for SOX7DIM/SOX18 as compared to SOX18 WT indicates that the dimerization is likely to be stabilized by secondary interactions outside the DIM domain that may be specific to SOX18.

Multiple sequence alignment of SOX18 DIM domain across 10 different species shows that the residues are mostly conserved throughout evolution (Figure 3C), especially in the region next to the third α-helix of the HMG domain (aa 161 to 168) as well as the FRELPPL motif, located in the last 17 amino acids preceding the TAD domain (aa 197–203). Further comparison of the DIM domain within the Human SOXF group reveals that the hydrophobic sequence FRELPPL is a specific feature of SOX18—the equivalent sequences in SOX7 and SOX17 are less hydrophobic—suggesting a potential role for this sequence in SOX18 homodimerization. To further investigate the role of this motif in SOX18/SOX18 interaction, we performed an AS assay between full length SOX18 and a deletion mutant that lacks the FRELPPL motif (Δ197–203). The deletion of this motif suppresses dimer formation (Supplementary Figure S4). In contrast, SOX18 FL was still able to form a dimer with the SOX18 deletion mutant lacking the first hydrophobic motif (Δ161–168) (Supplementary Figure S4). The DIM domain is a novel and unique feature of SOX18, with key hydrophobic motifs involved in the homodimerization process.

### A SOX18 homodimer binding motif is present on the chromatin

**Figure 4.**
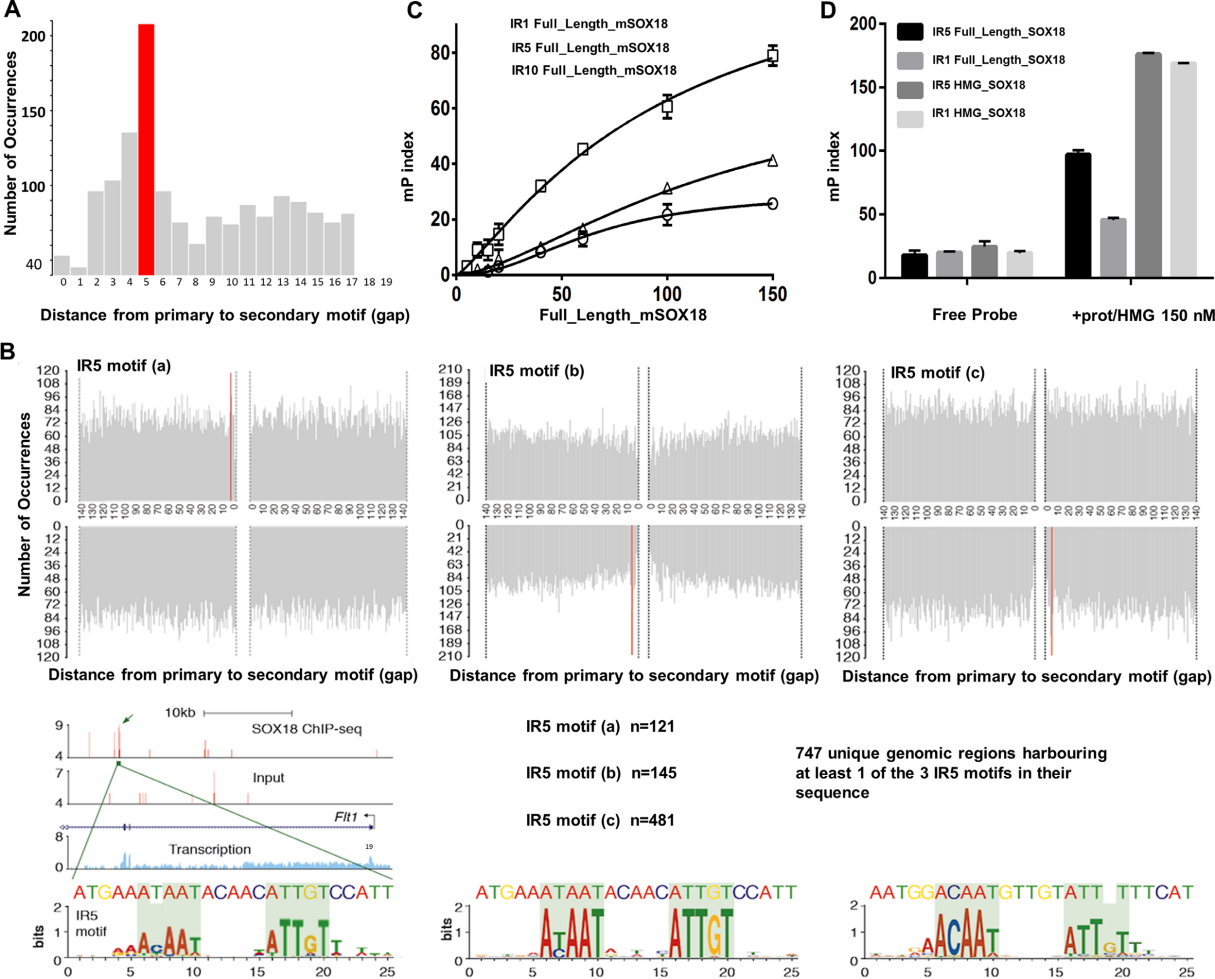
Identification of a consensus sequence for the homodimer of SOX18. (**A**) Spaced Motif Analysis Tool (SpaMo, MEME suite) analysis on SOX18 ChIP-seq data performed in HUVEC, showing the frequency of a secondary SOX18 motif in proximity of a primary SOX18 ChIP-seq peak at any given distance (1–140 bp). Enrichment for a 5-nucleotide spacer between a SOX inverted motif is shown in red (IR5, *P* = 0.005). (**B**) The four quadrants correspond to the strand orientation and upstream/downstream position relative to the ChIP-seq peak. An IR5 was identified for motif combination SOX18-SRY (IR5a), SRY-SRY (IR5b) and SOX18-SOX18 (IR5c). USCS browser track shows representative ChIP-seq peak in the FLT1 gene containing a SOX18 IR5a motif. Position weight matrixes show the inferred motifs based on the peaks containing the IR5a/b/c motifs. (**C**) Fluorescence polarization analysis of full-length SOX18 binding to FITC-labelled oligonucleotides harboring palindromic SOX18 binding motif identified in (B), separated by a 1 (IR1), 5 (IR5) or 10 (IR10) nucleotide spacer. Higher mP index indicates that SOX18 binds more favorably to IR5. (**D**) Fluorescence polarization analysis of full-length SOX18 and SOX18 HMG box construct binding to FITC-labelled oligonucleotides harboring palindromic SOX18 binding motif identified in (B), separated by a 5 (IR5) or 1 (IR1) nucleotide spacer (Supplementary Figure S2). Only IR5 and not IR1 can accommodate the dimer of full-length SOX18, but both can bind as efficiently to the HMG box.

In order to find a trace of the SOX18 dimer in the genome, we investigated the presence of a dimer-binding motif on the chromatin. To this end, we applied a motif based sequence analysis tool, Spaced Motif Analysis (SpaMo) (48), to search for an enrichment of a secondary SOX motif on the chromatin at a fixed distance from genomic SOX18 binding sites. We analysed the spacing between primary SOX18 binding sites and a putative secondary SOX site on the reported 23,635 peaks from the SOX18 ChIP-seq analysis (35), and identified a signature dimer motif that corresponds to a palindrome of the archetypical SOX motif 5′-AACAAT-3′, spaced by 5 nucleotides (Inverted repeat 5, IR5, *P*-value = 0.005) (Figure 4A, B). Since SOX proteins have a highly conserved HMG box and a very similar consensus-binding motif (5′-AACAAT-3′ or the reverse complement 5′-ATTGTT-3′), the spacing enrichment was identified for three combinations of SOX motifs: SOX18-SRY (IR5a), SOX18-SOX18 (IR5b) and SRY-SRY (IR5c), all corresponding to the inferred motif 5′-AC/TAATnnnnnATTGT-3′ (Figure 4B).

The IR5 motif closely resembles known dimer motifs identified for SOXE proteins such as SOX9^25,26^. Electrophoretic mobility shift assay (EMSA) experiments demonstrated that two SOX18-HMG domains, as well as two SOX9-HMG domains could simultaneously bind to this IR5 motif (Supplementary Figure S5).

To further confirm this observation, we took advantage of a fluorescence polarisation (FP) assay using FAM-labelled oligonucleotides harbouring IR motifs with different spacer lengths (IR1: 1 bp, IR5: 5 bp and IR10: 10 bp). In this assay, as proteins bind to DNA, the increase in molecular weight, as the protein-DNA complex forms, is reflected by an increase in the FP index (mP). This approach revealed that SOX18 full-length protein produces a maximum binding activity (higher mP index) in presence of an IR5 binding site (Figure 4C). There is approximately twice as much occupancy of SOX18 full-length on a probe that contains an IR5 motif, compared to one that has an IR1 motif (Figure 4C), since steric hindrance prevents cohabitation when the spacer is shorter. Occupancy on an IR1 probe could be restored to levels similar to those seen for an IR5 probe by using a SOX18-HMG fragment (aa 1–109), which allows for more physical overlap (Figure 4D).

### Sox18 dimerization is not simply a juxtaposition event on the DNA

**Figure 5.**
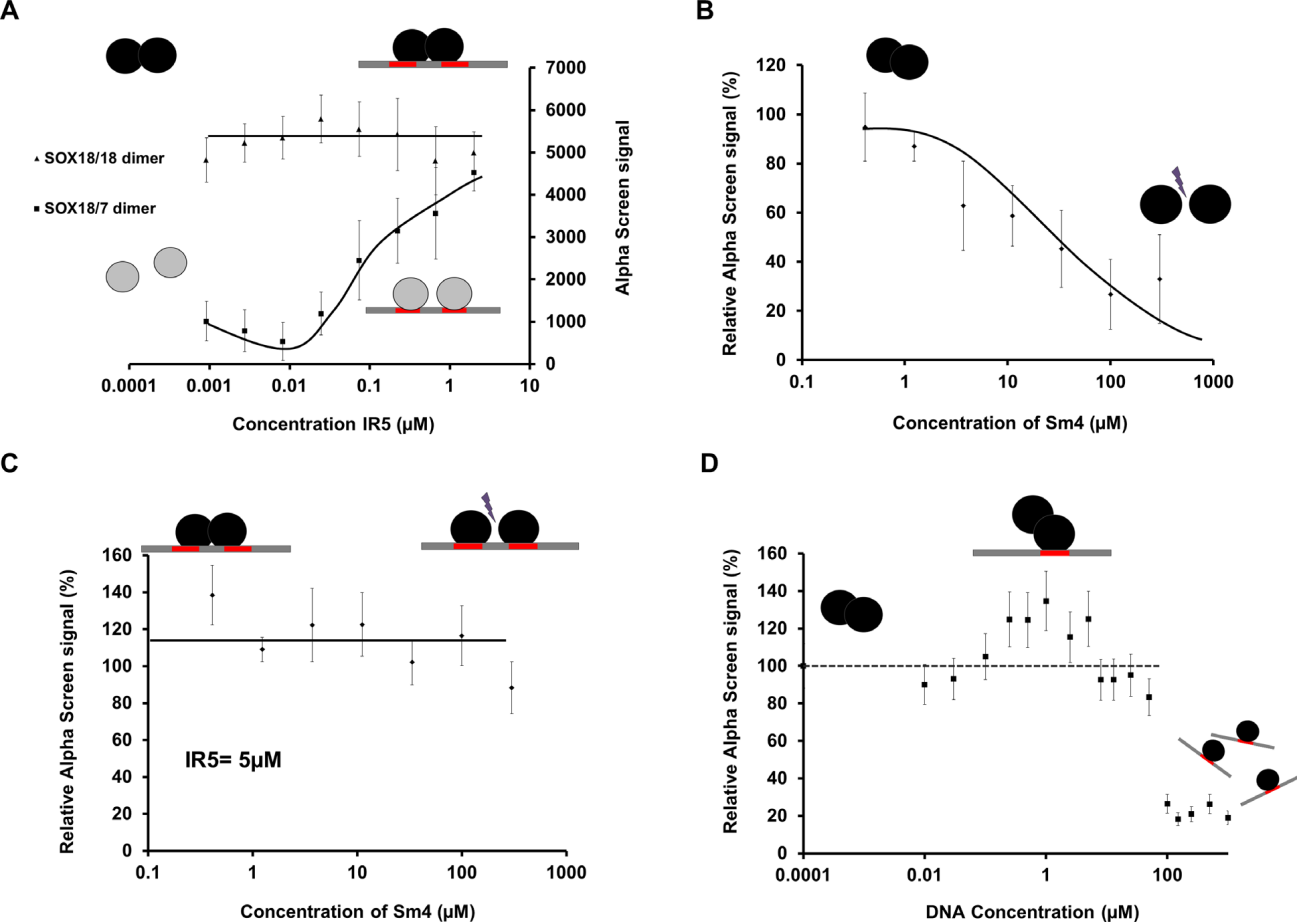
SOX18 dimerization on IR5 is not a juxtaposition event. In all cases, the black circles represent SOX18 WT, the grey circles correspond to the SOX18DIM/SOX7 swap constructs, the straight black line represents the double-stranded DNA on which the red rectangles are single consensus sequences. (**A**) Maximum AlphaScreen signal for the SOX18 constructs as a function of the consensus sequence IR5 concentration (μM). Addition of the IR5 motif in the SOX18 WT assay produced a minor change in the signal until saturation of the DNA was reached. In contrast, with the SOX18DIM/SOX7-swap mutant, as the concentration of IR5 increased, the AS signal steadily increased to reach the signal observed with the SOX18 WT. Note that the signal is restored to the level of SOX18 WT for IR5 concentration >1.5 μM. (**B**) Maximum AlphaScreen signal for SOX18 WT as a function of concentration of **Sm4. Sm4** significantly disrupts the SOX18 homodimer in the absence of IR5 motif-containing DNA, with IC_50_ value around 3 μM (35). (**C**) Maximum AlphaScreen signal for SOX18 WT as a function of concentration of **Sm4** and in the presence of a fixed concentration of IR5-containing DNA (5 μM). At this DNA concentration, the AS signal intensity was not perturbed upon addition of Sm4 up to 100μM. (**D**) Maximum AlphaScreen signal for SOX18 WT as a function of concentration of single DNA consensus sequence (μM). As the DNA concentration increases, the AS signal intensity increases slightly until 1μM then the signal decreases as SOX18 binds to individual DNA strands.

In order to tease apart a cooperative binding mechanism from a co-binding event that does not involve a PPI, we performed AS experiments using SOX18 and the SOX18DIM/SOX7-swap mutant in presence of an oligonucleotide harbouring the IR5 palindromic sequence. The lack of dimerization capability of the mutant protein only allows monomeric binding. Incubation of the IR5 probe in presence of SOX18 reaches a plateau phase almost instantly with only a mild increase of the maximum AS signal observed. In contrast, the SOX18DIM/SOX7-swap mutant responded in a dose-dependent manner to an increase of the IR5 probe concentration (Figure 5A). The main difference between the wild type and the mutant protein lies in their abilities to elicit protein-protein interactions, and in particular homo-dimer formation.

Next, we evaluated the effects of pharmacologically disrupting SOX18 dimer formation in this context. The small molecule inhibitor **Sm4** interferes with SOX18-dependent PPIs, including its homodimerization (35). As previously described, **Sm4** significantly disrupted the SOX18 homodimer in absence of IR5 motif-containing oligonucleotide with an IC_50_ value around 3μM (Figure 5B). However, in presence of the IR5 probe, the AS signal intensity was unperturbed upon addition of **Sm4** at up to 100 μM (Figure 5C). This suggests that despite disruption of the SOX18 dimer formation caused by **Sm4**, two SOX18 monomers can still co-bind to the IR5 motif and produce AS signal, in a similar fashion to the SOX18 DIM/SOX 7 swap protein.

Finally, when AS was performed in the presence of DNA harbouring a single consensus SOX binding motif, we observed a small increase of the signal strength as the concentration of probe increases (to 1 μM) until all dimers are displaced by binding to individual DNA probes (>5 μM) (Figure 5D). This effect is specific to the SOX18 dimer (Supplementary Figure S6). Taken together, these experiments show that formation of the SOX18 dimer does not require the presence of IR5 (contrary to SOX18DIM/SOX7) even though the dimer can be stabilized by the presence of DNA.

### SOX18 dimer has an endothelial specific signature

Analysis of the SOX18 ChIP-seq data set revealed 747 unique genomic regions harbouring at least one of the three IR5 motifs in their sequence. The IR5 motif was identified scanning for a more or less relaxed secondary SOX binding site in the vicinity of a primary SOX motif. We chose 3 different combinations of motifs since the consensus binding sequence for SOX proteins is short and often degenerated (49). To be exhaustive, we considered the following variations: SOX18-SRY (IR5a), SOX18-SOX18 (IR5b) and SRY-SRY (IR5c). Genomic regions enrichment of annotations tool (GREAT) (50) analysis of the genome-wide distribution of the SOX18 ChIP-seq peaks containing an IR5a-c motif assigned to these sequences a total of 964 genes. Genotype-Tissue Expression (GTEx) analysis of this gene list revealed that about one-third of them are significantly expressed by endothelial cells (Supplementary Figure S7). In particular, some putative regulatory sequences containing an IR5a-c motif have been assigned to specific vascular endothelial markers that include, but is not limited to, *FLT1, Endomucin, SEMA3D, MEF2A, MAP4K4* and *NRP1*, as well as other genes known to be involved in angiogenesis such as *IL33* and *KLF4* (Supplementary Figure S7). Further analysis of SOX18 ChIP-seq peaks containing IR5a-c motif using EpiExplorer software (51) enabled us to define the overlap with histone marks and DNase hypersensitivity regions publically available from the ENCODE consortium (Supplementary Figures S8-S9 and Supplementary Table S2). A large portion of the peaks intersect with active regulatory regions of the HUVEC genome, with 371 regions showing at least 50% overlap with no less than two histone marks for active transcription. Conversely, some IR5 motifs (∼50%) overlap with at least one repressive mark (H3K27me3 or H3K36me3) (Supplementary Figure S8A). This observation indirectly suggests that the SOX18 dimer has the potential to act as both a repressor and an activator of transcription. This capability is likely to be modulated by protein partner recruitment and different cell subtype.

**Figure 6.**
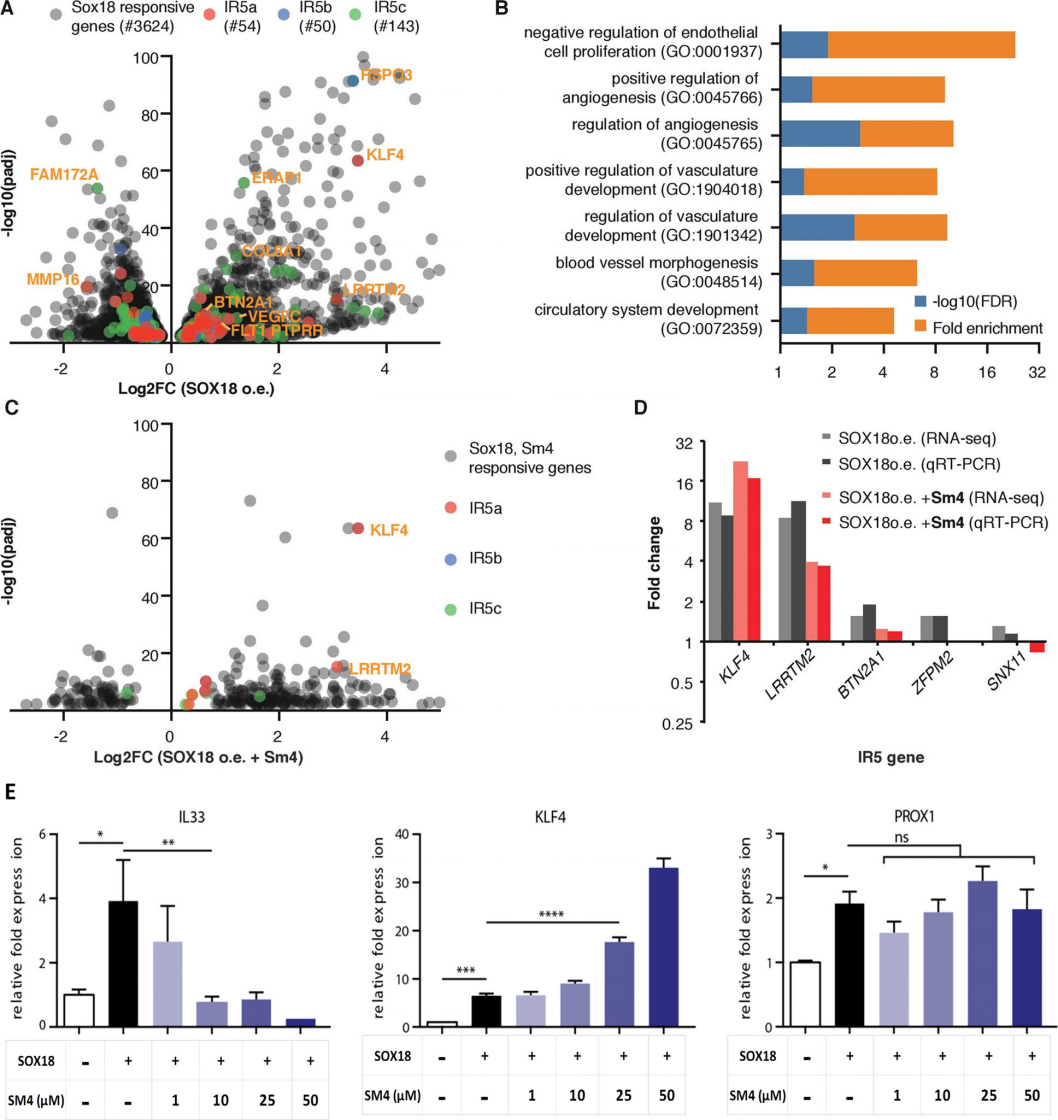
Transcriptional endothelial signature of the SOX18 homodimer. (**A**) Volcano plot showing genes responsive to SOX18 over-expression (o.e.) in HUVECs. Genes with an IR5 motif have been highlighted in red (IR5a), blue (IR5b) and green (IR5c) and a selection of highly responsive genes has been annotated (e.g. *KLF4*). (**B**) GO term analysis (molecular function, PANTHER v13.1 (67)) filtered for vascular and endothelial terms for SOX18 responsive IR5 genes, revealing significant enrichment of up to 21.3 fold (Fisher test, FDR < 0.05). More comprehensive GO analysis for IR5 vs. non IR5 genes is presented in Supplementary Figure S10. (**C**) Volcano plot showing genes responsive to both SOX18 o.e. and to treatment with inhibitor **Sm4**. IR5 genes are labelled as in (A). Most **Sm4** responsive IR5 genes are positively regulated by SOX18, including *KLF4* and *LRRTM2*. (**D**) Validation of IR5 gene regulation using representative SOX18, **Sm4** responsive IR5 genes. Data from qRT-PCR matches RNA-seq results and shows positive relation of IR5 genes by SOX18 o.e. and impact of SOX18 inhibition. (**E**) Dose response of **Sm4** treatment (1–50 μM) by qRT-PCR analysis shows inhibition or over-expression of *IL33* and *KLF4* transcripts, respectively. *PROX1* mRNA was not significantly affected. Error bars are s.e.m.

To further assess the functional relevance of the SOX18 dimer in endothelial cells, we took advantage of a previously published RNA-seq dataset where SOX18 was overexpressed in HUVECs, in presence and absence of the small molecule inhibitor **Sm4** (35). The over-expression of SOX18 caused a broad range of genes to be up- or down-regulated (3621, 53% up) (Figure 6A, grey dots, Supplementary Figure S10A). GO analysis showed enrichment for biological processes involved in angiogenesis (1.67-fold, FDR < 0.01), hematopoiesis (1.52-fold, FDR < 0.01) and wound healing (1.44-fold, FDR < 0.05), typical SOX18 functions (Supplementary Figure S10B).

This list of SOX18-responsive genes was then cross-referenced to the list of genes associated to IR5a–c motifs in order to ascertain which of the putative dimer genes would be most likely to be biologically relevant in an endothelial cell context. We found a set of 261 genes that met these criteria, being both responsive to SOX18 overexpression and having at least one of the 3 IR5 motifs in their putative regulatory elements (Figure 6A, red/blue/green dots). GO analysis of this gene subset revealed a strong enrichment for endothelial and angiogenic terms within biological processes, particularly negative regulation of endothelial cell proliferation (21.3-fold enrichment, FDR < 0.05), positive regulation of angiogenesis (7.49-fold, FDR < 0.05) and positive regulation of vascular development (6.78-fold, FDR < 0.05) (Figure 6B). The enrichment for these terms in the IR5 gene set was much higher than in the non-filtered set of SOX18 responsive genes. This suggests that the non-dimeric and the dimeric forms are involved in distinct biological processes (Supplementary Figure S10C).

To further validate these findings, we analysed the effect of the protein-protein interaction disruptor **Sm4** on SOX18 responsive genes (Figure 6C). Several dimer genes were affected by **Sm4**; interestingly, 90% of those genes were also positively regulated by SOX18 overexpression. **Sm4** strongly affected a subset of IR5 genes, highlighted in Figure 6C. A full list of the IR5 genes affected by **Sm4** is provided in Supplementary Table S3. Dysregulation of gene expression was further profiled by qRT-PCR analysis (Figure 6D). Results validated genome-wide overlapping analysis with histone marks, suggesting that the dimer has the capability to activate or repress transcription, since the small molecule inhibitor was able to enhance or repress gene expression. Lastly, the effect of **Sm4** on the transcriptional activity of SOX18 was interrogated in further details for known key endothelial regulators such as *IL33, KLF4* or *PROX1* (Figure 6E). **Sm4** selectively caused a SOX18-dependent dose response on the expression of genes harbouring an IR5 motif (*IL33* and *KLF4*) (Figure 6E). SOX18-dependent *IL33* activation was inhibited by **Sm4**, whereas *KLF4* activation was enhanced. In contrast, *PROX1*, a known SOX18 target gene that only contains monomer motifs in its regulatory region of intron 1, was not significantly affected by **Sm4** treatment (Figure 6E). These results show that the SOX18 dimer has a distinct molecular role from the monomer and selectively regulates a subset of endothelial specific genes that are likely to be context dependent.

## DISCUSSION

Here, we describe the molecular basis for the dimerization of the SOX18 transcription factor, a key player during endothelial cell fate determination. We quantitatively describe this homotypic interaction, uniquely observed within the SOXF group and demonstrate the existence and functional relevance of SOX18 homodimer, showing the presence of an homodimer signature in the genome and controlling gene expression using pharmacological interferences with a small molecule inhibitor.

In humans, 20 Sry-related high-mobility-group box (SOX) genes have been identified, characterised, and categorised into 8 groups (29) (A-H). Across all SOX proteins, the HMG-box is highly conserved. In contrast, protein regions outside this DNA-binding domain (52) are highly variable in length and amino-acid composition. The HMG-box is thought to be central to target gene selectivity via both specific DNA motif recognition and protein partner recruitment. The functional consequences of switching the HMG-box between SOX2 and SOX17 have been shown to affect endodermal programing, by altering enhancer selection in combination with differential recruitment of OCT4 (53). In recent years, it became apparent that the domains outside of the HMG-box also contribute to protein partner recruitment.

Only a handful of SOX protein have been shown to dimerize (see for review (14,16)) even though the high throughput SELEX approach has predicted that most of SOX TFs are likely to form homodimers (54). SOX dimerization behaviours fall into three distinct groups. Some SOXs, such as the ones in the E Group (SOX8, SOX9, and SOX10), homodimerize in a DNA-dependent manner. SOXE proteins encode a unique 40 amino acids dimerization (DIM) domain which precedes the HMG-box (55). SOXE TFs dimerize in a highly cooperative fashion, but only do so in the presence of a (A/T)(A/T)CAA(A/T)G palindromic DNA binding sequence (56–58). Dimers of SOXE factors are able to accommodate a range of variably spaced half-sites (30,59), as opposed to other TFs that favour composite DNA elements with fixed spacing. All three SOXE proteins also effectively heterodimerize with one another, but do not dimerize with non-SOXE proteins. Interestingly, truncated DIM-SOXE fragments can also effectively dimerize with isolated SOXE HMG boxes, suggesting that a single SOXE group DIM domain is necessary and sufficient to mediate dimer formation. In this process, the dimerization is driven in the main by DIM-HMG intramolecular interactions communicated to the HMG of the juxtaposed SOX protein rather than by direct DIM–DIM intermolecular interactions. In contrast, for SOX18 the presence of two DIM domains seems to be mandatory for dimer assembly. Indeed, we show that SOX18 WT and the SOX18 mutant (minus FRELPPL motif, Δ197-203) are not able to form a dimer (Supplementary Figure S4).

SOX2 is another protein able to form a dimer in a DNA-dependent fashion. It has been shown that both monomeric and dimeric forms are present in human neutrophils (60). The dimerization propensity of SOX2 has been validated at a transcriptional level whereby the dimerization of SOX2 is triggered by the presence of bacterial DNA, and unlike the monomeric form, activates the TAB2-TAK1 complex, leading to the stimulation of the innate immune response (61). As in SOX18, the Group B homolog (GBH) domain required for SOX2 dimerization is at the C-terminus of SOX2 HMG-box.

In contrast to the DNA-dependent dimerization processes of the SOXE, members of the D-Group (SOX5/SOX6/SOX13) are known for dimerizing via a leucine zipper motif in a DNA-independent manner (62). A coiled-coil domain mediates homo- and heterotypic interactions within the SOXD group (63). This dimerization domain is situated in the N-terminal part of the protein and enables cooperative binding to clustered SOX-responsive elements (64). Our study supports the idea that regions located outside the HMG-box play an essential role in the dimerization process since we locate the SOX18 DIM domain within a unique 50 amino acids region adjacent to the 3^rd^ α-helix of the HMG-box. This localization is in good agreement with our previous observation that binding of an antibody raised against the 3rd α-helix of the HMG-box prevents homodimerization^14^. As with the SOXD group, we speculate that the self-assembly process of SOX18 might be DNA-independent, since dimerization occurs both in the in presence or absence of an IR5 oligonucleotide.

As for SOX2, SOX9, and SOX10, a subset of SOX-responsive genes are specifically regulated by SOX18 dimer activity. In the case of SOXE proteins, dimerization partially drives transcriptional output specificity. For instance, SOX10 homodimer binding sites are found in enhancers of several SOX10 target genes, including connexin-32, protein zero and myelin basic protein. Occupation of both SOX binding sites is required to drive promoter activities (65). SOX10 dimers also influence the formation of multi-protein complexes and transcriptional activity from these promoters (57). SOX9 homodimer-binding sequences are found in the enhancers of collagen and it has been shown that the SOX9 dimer recruits SOX5/6 dimers to activate *Col2a1* transcription. In a similar fashion, we show that the SOX18 dimer has the capability to recruit the *notch* effector RBPJ or the transcription factor MEF2C (this study, Figure 1F) to probably further regulate transcription of dimer responsive genes (35,66). In most cases, it seems that the presence of a non-compact SOX-binding motif is a good marker to track potential transcriptional regulation by a dimer. In the case of SOX18, and in contrast to SOXE proteins, the spacer size is critical for cooperative binding and is found mainly in enhancer regions located 50Kb to 500Kb from gene transcription start sites.

In conclusion, structural and functional variations within different members of the SOX family make the identification and characterisation of the dimerization process a tedious exercise. Different modalities of self-assembly, involving the DNA, the HMG-box and specific motifs in C-terminal and N-terminal positions outside the DNA-binding domain, contribute to the diversity of self-assembly mechanisms. Our work shows that the mechanism of SOX18 dimer formation is a unique feature within the F-group, and involves a distinct binding motif, which permits the transcriptional signature of SOX18 to be distinguished from confounding, closely related, and redundant, SOX7 and SOX17 activities.

## Supplementary Material

Supplementary Data

## References

[1] De Val S., Black B.L. Transcriptional control of endothelial cell development. Dev. Cell. 2009; 16:180–195. 19217421 10.1016/j.devcel.2009.01.014 PMC2728550

[2] Levine M., Davidson E.H. Gene regulatory networks for development. PNAS. 2005; 102:4936–4942. 15788537 10.1073/pnas.0408031102 PMC555974

[3] MacQuarrie K.L., Fong A.P., Morse R.H., Tapscott S.J. Genome-wide transcription factor binding: beyond direct target regulation. Trends Genet. 2011; 27:141–148. 21295369 10.1016/j.tig.2011.01.001 PMC3068217

[4] Bowles J., Schepers G., Koopman P. Phylogeny of the SOX family of developmental transcription factors based on sequence and structural indicators. Dev. Biol. 2000; 227:239–255. 11071752 10.1006/dbio.2000.9883

[5] Wilson M., Koopman P. Matching SOX: partner proteins and co-factors of the SOX family of transcriptional regulators. Curr. Opin. Genet. Dev. 2002; 12:441–446. 12100890 10.1016/s0959-437x(02)00323-4

[6] Wegner M. From head to toes: the multiple facets of Sox proteins. Nucleic Acids Res. 1999; 27:1409–1420. 10037800 10.1093/nar/27.6.1409 PMC148332

[7] Schepers G.E., Teasdale R.D., Koopman P. Twenty pairs of sox: extent, homology, and nomenclature of the mouse and human sox transcription factor gene families. Dev. Cell. 2002; 3:167–170. 12194848 10.1016/s1534-5807(02)00223-x

[8] Francois M., Koopman P., Beltrame M. SoxF genes: Key players in the development of the cardio-vascular system. Int. J. Biochem. Cell Biol. 2010; 42:445–448. 19733255 10.1016/j.biocel.2009.08.017

[9] Kent J., Wheatley S.C., Andrews J.E., Sinclair A.H., Koopman P. A male-specific role for SOX9 in vertebrate sex determination. Development. 1996; 122:2813–2822. 8787755 10.1242/dev.122.9.2813

[10] Bi W., Deng J.M., Zhang Z., Behringer R.R., de Crombrugghe B. Sox9 is required for cartilage formation. Nat. Genet. 1999; 22:85–89. 10319868 10.1038/8792

[11] Wegner M. SOX after SOX: SOXession regulates neurogenesis. Genes Dev. 2011; 25:2423–2428. 22156204 10.1101/gad.181487.111 PMC3243053

[12] Masui S., Nakatake Y., Toyooka Y., Shimosato D., Yagi R., Takahashi K., Okochi H., Okuda A., Matoba R., Sharov A.A. Pluripotency governed by Sox2 via regulation of Oct3/4 expression in mouse embryonic stem cells. Nat. Cell Biol. 2007; 9:625–635. 17515932 10.1038/ncb1589

[13] Fontaine F.R., Goodall S., Prokop J.W., Howard C.B., Moustaqil M., Kumble S., Rasicci D.T., Osborne G.W., Gambin Y., Sierecki E. mAbs. 2018; 10:Taylor & Francis 596–606. 29648920 10.1080/19420862.2018.1451288 PMC5972640

[14] Sarkar A., Hochedlinger K. The sox family of transcription factors: versatile regulators of stem and progenitor cell fate. Cell Stem Cell. 2013; 12:15–30. 23290134 10.1016/j.stem.2012.12.007 PMC3608206

[15] Polanco J.C., Koopman P. Sry and the hesitant beginnings of male development. Dev. Biol. 2007; 302:13–24. 16996051 10.1016/j.ydbio.2006.08.049

[16] Kamachi Y., Kondoh H. Sox proteins: regulators of cell fate specification and differentiation. Development. 2013; 140:4129–4144. 24086078 10.1242/dev.091793

[17] Irrthum A., Devriendt K., Chitayat D., Matthijs G., Glade C., Steijlen P.M., Fryns J.-P., Van Steensel M.A., Vikkula M. Mutations in the transcription factor gene SOX18 underlie recessive and dominant forms of hypotrichosis-lymphedema-telangiectasia. Am. J. Hum. Genet. 2003; 72:1470–1478. 12740761 10.1086/375614 PMC1180307

[18] Corada M., Orsenigo F., Morini M.F., Pitulescu M.E., Bhat G., Nyqvist D., Breviario F., Conti V., Briot A., Iruela-Arispe M.L. Sox17 is indispensable for acquisition and maintenance of arterial identity. Nat. Commun. 2013; 4:2609. 24153254 10.1038/ncomms3609 PMC3826640

[19] François M., Caprini A., Hosking B., Orsenigo F., Wilhelm D., Browne C., Paavonen K., Karnezis T., Shayan R., Downes M. Sox18 induces development of the lymphatic vasculature in mice. Nature. 2008; 456:643–647. 18931657 10.1038/nature07391

[20] Matsui T., Kanai-Azuma M., Hara K., Matoba S., Hiramatsu R., Kawakami H., Kurohmaru M., Koopman P., Kanai Y. Redundant roles of Sox17 and Sox18 in postnatal angiogenesis in mice. J. Cell Sci. 2006; 119:3513–3526. 16895970 10.1242/jcs.03081

[21] Pennisi D., Gardner J., Chambers D., Hosking B., Peters J., Muscat G., Abbott C., Koopman P. Mutations in Sox18 underlie cardiovascular and hair follicle defects in ragged mice. Nat. Genet. 2000; 24:434–437. 10742113 10.1038/74301

[22] Moalem S., Brouillard P., Kuypers D., Legius E., Harvey E., Taylor G., Francois M., Vikkula M., Chitayat D. Hypotrichosis‐lymphedema‐telangiectasia‐renal defect associated with a truncating mutation in the SOX18 gene. Clin. Genet. 2015; 87:378–382. 24697860 10.1111/cge.12388

[23] Valenzuela I., Fernández-Alvarez P., Plaja A., Ariceta G., Sabaté-Rotés A., García-Arumí E., Vendrell T., Tizzano E. Further delineation of the SOX18-related Hypotrichosis, Lymphedema, Telangiectasia syndrome (HTLS). Eur. J. Med. Genet. 2018; 61:269–272. 29307792 10.1016/j.ejmg.2018.01.001

[24] James K., Hosking B., Gardner J., Muscat G.E., Koopman P. Sox18 mutations in the ragged mouse alleles ragged‐like and opossum. Genesis. 2003; 36:1–6. 12748961 10.1002/gene.10190

[25] Hosking B., François M., Wilhelm D., Orsenigo F., Caprini A., Svingen T., Tutt D., Davidson T., Browne C., Dejana E. Sox7 and Sox17 are strain-specific modifiers of the lymphangiogenic defects caused by Sox18 dysfunction in mice. Development. 2009; 136:2385–2391. 19515696 10.1242/dev.034827

[26] Xenarios I., Salwinski L., Duan X.J., Higney P., Kim S.-M., Eisenberg D. DIP, the Database of Interacting Proteins: a research tool for studying cellular networks of protein interactions. Nucleic Acids Res. 2002; 30:303–305. 11752321 10.1093/nar/30.1.303 PMC99070

[27] Alm E., Arkin A.P. Biological networks. Curr. Opin. Struct. Biol. 2003; 13:193–202. 12727512 10.1016/s0959-440x(03)00031-9

[28] Oettgen P. Transcriptional regulation of vascular development. Circ. Res. 2001; 89:380–388. 11532898 10.1161/hh1701.095958

[29] Lefebvre V., Dumitriu B., Penzo-Méndez A., Han Y., Pallavi B. Control of cell fate and differentiation by Sry-related high-mobility-group box (Sox) transcription factors. Int. J. Biochem.Cell Biol. 2007; 39:2195–2214. 17625949 10.1016/j.biocel.2007.05.019 PMC2080623

[30] Huang Y.-H., Jankowski A., Cheah K.S., Prabhakar S., Jauch R. SOXE transcription factors form selective dimers on non-compact DNA motifs through multifaceted interactions between dimerization and high-mobility group domains. Sci. Rep. 2015; 5:10398. 26013289 10.1038/srep10398 PMC4445065

[31] Akiyama H., Chaboissier M.-C., Martin J.F., Schedl A., de Crombrugghe B. The transcription factor Sox9 has essential roles in successive steps of the chondrocyte differentiation pathway and is required for expression of Sox5 and Sox6. Genes Dev. 2002; 16:2813–2828. 12414734 10.1101/gad.1017802 PMC187468

[32] Akiyama H., Chaboissier M.-C., Behringer R.R., Rowitch D.H., Schedl A., Epstein J.A., de Crombrugghe B. Essential role of Sox9 in the pathway that controls formation of cardiac valves and septa. PNAS. 2004; 101:6502–6507. 15096597 10.1073/pnas.0401711101 PMC404074

[33] Foster J., Dominguez-Steglich M., Guioli S., Kowk G., Weller P., Stevanovic M., Weissenbach J., Mansour S., Young I.D., Goodfellow P. Campomelic dysplasia and autosomal sex reversal caused by mutations in an Sry-related gene. Nature. 1994; 372:525–530. 7990924 10.1038/372525a0

[34] Wagner T., Wirth J., Meyer J., Zabel B., Held M., Zimmer J., Pasantes J., Bricarelli F.D., Keutel J., Hustert E. Autosomal sex reversal and campomelic dysplasia are caused by mutations in and around the SRY-related gene SOX9. Cell. 1994; 79:1111–1120. 8001137 10.1016/0092-8674(94)90041-8

[35] Overman J., Fontaine F., Moustaqil M., Mittal D., Sierecki E., Sacilotto N., Zuegg J., Robertson A.A., Holmes K., Salim A.A. Pharmacological targeting of the transcription factor SOX18 delays breast cancer in mice. eLife. 2017; 6:e21221. 28137359 10.7554/eLife.21221 PMC5283831

[36] Gagoski D., Mureev S., Giles N., Johnston W., Dahmer-Heath M., Skalamera D., Gonda T.J., Alexandrov K. Gateway-compatible vectors for high-throughput protein expression in pro- and eukaryotic cell-free systems. J. Biotechnol. 2015; 195:1–7. 25529348 10.1016/j.jbiotec.2014.12.006

[37] Sierecki E., Giles N., Polinkovsky M., Moustaqil M., Alexandrov K., Gambin Y. A cell-free approach to accelerate the study of protein-protein interactions in vitro. Interface Focus. 2013; 3:20130018. 24511386 10.1098/rsfs.2013.0018 PMC3915825

[38] Kovtun O., Mureev S., Jung W., Kubala M.H., Johnston W., Alexandrov K. Leishmania cell-free protein expression system. Methods. 2011; 55:58–64. 21704167 10.1016/j.ymeth.2011.06.006

[39] Mureev S., Kovtun O., Nguyen U.T., Alexandrov K. Species-independent translational leaders facilitate cell-free expression. Nat. Biotechnol. 2009; 27:747–752. 19648909 10.1038/nbt.1556

[40] Sierecki E., Stevers L.M., Giles N., Polinkovsky M.E., Moustaqil M., Mureev S., Johnston W.A., Dahmer-Heath M., Skalamera D., Gonda T.J. Rapid mapping of interactions between Human SNX-BAR proteins measured in vitro by AlphaScreen and single-molecule spectroscopy. Mol. Cell. Proteomics: MCP. 2014; 13:2233–2245. 24866125 10.1074/mcp.M113.037275 PMC4159646

[41] Gambin Y., Schug A., Lemke E.A., Lavinder J.J., Ferreon A.C.M., Magliery T.J., Onuchic J.N., Deniz A.A. Direct single-molecule observation of a protein living in two opposed native structures. Proc. Natl. Acad. Sci. U.S.A. 2009; 106:10153–10158. 19506258 10.1073/pnas.0904461106 PMC2700882

[42] Ng C.K., Li N.X., Chee S., Prabhakar S., Kolatkar P.R., Jauch R. Deciphering the Sox-Oct partner code by quantitative cooperativity measurements. Nucleic Acids Res. 2012; 40:4933–4941. 22344693 10.1093/nar/gks153 PMC3367189

[43] Gambin Y., Polinkovsky M., Francois B., Giles N., Bhumkar A., Sierecki E. Confocal spectroscopy to study dimerization, oligomerization and aggregation of Proteins: A practical guide. Int. J. Mol. Sci. 2016; 17:655. 10.3390/ijms17050655 PMC4881481 27144560

[44] Bernard P., Tang P., Liu S., Dewing P., Harley V.R., Vilain E. Dimerization of SOX9 is required for chondrogenesis, but not for sex determination. Hum. Mol. Genet. 2003; 12:1755–1765. 12837698 10.1093/hmg/ddg182

[45] Hosking B.M., Wang S.M., Chen S.L., Penning S., Koopman P., Muscat G.E. SOX18 directly interacts with MEF2C in endothelial cells. Biochem. Biophys. Res. Commun. 2001; 287:493–500. 11554755 10.1006/bbrc.2001.5589

[46] Hu C.-D., Kerppola T.K. Simultaneous visualization of multiple protein interactions in living cells using multicolor fluorescence complementation analysis. Nat. Biotechnol. 2003; 21:539–545. 12692560 10.1038/nbt816 PMC1820765

[47] Harvey S.A., Smith J.C. Visualisation and quantification of morphogen gradient formation in the zebrafish. PLoS Biol. 2009; 7:e1000101. 19419239 10.1371/journal.pbio.1000101 PMC2675906

[48] Whitington T., Frith M.C., Johnson J., Bailey T.L. Inferring transcription factor complexes from ChIP-seq data. Nucleic Acids Res. 2011; 39:e98. 21602262 10.1093/nar/gkr341 PMC3159476

[49] Klaus M., Prokoph N., Girbig M., Wang X., Huang Y.-H., Srivastava Y., Hou L., Narasimhan K., Kolatkar P.R., Francois M. Structure and decoy-mediated inhibition of the SOX18/Prox1-DNA interaction. Nucleic Acids Res. 2016; 44:3922–3935. 26939885 10.1093/nar/gkw130 PMC4856986

[50] McLean C.Y., Bristor D., Hiller M., Clarke S.L., Schaar B.T., Lowe C.B., Wenger A.M., Bejerano G. GREAT improves functional interpretation of cis-regulatory regions. Nat. Biotechnol. 2010; 28:495. 20436461 10.1038/nbt.1630 PMC4840234

[51] Halachev K., Bast H., Albrecht F., Lengauer T., Bock C. EpiExplorer: live exploration and global analysis of large epigenomic datasets. Genome Biol. 2012; 13:R96. 23034089 10.1186/gb-2012-13-10-r96 PMC3491424

[52] Sekido R. SRY: A transcriptional activator of mammalian testis determination. Int. J. Biochem. Cell Biol. 2010; 42:417–420. 20005972 10.1016/j.biocel.2009.12.005

[53] Aksoy I., Jauch R., Chen J., Dyla M., Divakar U., Bogu G.K., Teo R., Ng C.K.L., Herath W., Lili S. Oct4 switches partnering from Sox2 to Sox17 to reinterpret the enhancer code and specify endoderm. EMBO J. 2013; 32:938–953. 23474895 10.1038/emboj.2013.31 PMC3616284

[54] Jolma A., Yan J., Whitington T., Toivonen J., Nitta K.R., Rastas P., Morgunova E., Enge M., Taipale M., Wei G. DNA-binding specificities of human transcription factors. Cell. 2013; 152:327–339. 23332764 10.1016/j.cell.2012.12.009

[55] Schlierf B., Ludwig A., Klenovsek K., Wegner M. Cooperative binding of Sox10 to DNA: requirements and consequences. Nucleic Acids Res. 2002; 30:5509–5516. 12490719 10.1093/nar/gkf690 PMC140074

[56] Sock E., Pagon R.A., Keymolen K., Lissens W., Wegner M., Scherer G. Loss of DNA-dependent dimerization of the transcription factor SOX9 as a cause for campomelic dysplasia. Hum. Mol. Genet. 2003; 12:1439–1447. 12783851 10.1093/hmg/ddg158

[57] Peirano R.I., Wegner M. The glial transcription factor Sox10 binds to DNA both as monomer and dimer with different functional consequences. Nucleic Acids Res. 2000; 28:3047–3055. 10931919 10.1093/nar/28.16.3047 PMC108444

[58] Harley V.R., Lovell-Badge R., Goodfellow P.N. Definition of a consensus DNA binding site for SRY. Nucleic Acids Res. 1994; 22:1500. 8190643 10.1093/nar/22.8.1500 PMC308012

[59] Jankowski A., Szczurek E., Jauch R., Tiuryn J., Prabhakar S. Comprehensive prediction in 78 human cell lines reveals rigidity and compactness of transcription factor dimers. Genome Res. 2013; 23:1307–1318. 23554463 10.1101/gr.154922.113 PMC3730104

[60] Xia P., Wang S., Ye B., Du Y., Huang G., Zhu P., Fan Z. Sox2 functions as a sequence-specific DNA sensor in neutrophils to initiate innate immunity against microbial infection. Nat. Immunol. 2015; 16:366–375. 25729924 10.1038/ni.3117

[61] Kamachi Y., Sockanathan S., Liu Q., Breitman M., Lovell-Badge R., Kondoh H. Involvement of SOX proteins in lens-specific activation of crystallin genes. EMBO J. 1995; 14:3510. 7628452 10.1002/j.1460-2075.1995.tb07357.x PMC394419

[62] Takamatsu N., Kanda H., Tsuchiya I., Yamada S., Ito M., Kabeno S., Shiba T., Yamashita S. A gene that is related to SRY and is expressed in the testes encodes a leucine zipper-containing protein. Mol. Cell. Biol. 1995; 15:3759–3766. 7791783 10.1128/mcb.15.7.3759 PMC230614

[63] Lefebvre V., Li P., De Crombrugghe B. A new long form of Sox5 (L‐Sox5), Sox6 and Sox9 are coexpressed in chondrogenesis and cooperatively activate the type II collagen gene. EMBO J. 1998; 17:5718–5733. 9755172 10.1093/emboj/17.19.5718 PMC1170900

[64] Connor F., Cary P.D., Read C.M., Preston N.S., Driscoll P.C., Denny P., Crane-Robinson C., Ashworth A. DNA binding and bending properties of the postmeiotically expressed Sry-related protein Sox-5. Nucleic Acids Res. 1994; 22:3339–3346. 8078769 10.1093/nar/22.16.3339 PMC523727

[65] Bondurand N., Girard M., Pingault V., Lemort N., Dubourg O., Goossens M. Human Connexin 32, a gap junction protein altered in the X-linked form of Charcot–Marie–Tooth disease, is directly regulated by the transcription factor SOX10. Hum. Mol. Genet. 2001; 10:2783–2795. 11734543 10.1093/hmg/10.24.2783

[66] Fontaine F., Overman J., Moustaqil M., Mamidyala S., Salim A., Narasimhan K., Prokoph N., Robertson A.A.B., Lua L., Alexandrov K. Small-Molecule inhibitors of the SOX18 transcription factor. Cell Chem. Biol. 2017; 24:346–359. 28163017 10.1016/j.chembiol.2017.01.003

[67] Mi H., Muruganujan A., Thomas P.D. PANTHER in 2013: modeling the evolution of gene function, and other gene attributes, in the context of phylogenetic trees. Nucleic Acids Res. 2012; 41:D377–D386. 23193289 10.1093/nar/gks1118 PMC3531194

