# Supplementary material for: Homodimerization regulates an endothelial specific signature of the SOX18 transcription factor
Source: Nucleic Acids Res. 2018 Oct 18;46(21):11381–95. doi: 10.1093/nar/gky897 (PMC6265484; doi:10.1093/nar/gky897)
Supplement: Supplementary Data [file gky897_supplemental_files.zip › New Moustaqil et al revision Suppl Info.pdf]

SUPPLEMENTARY INFORMATION

Figure S1: Cell Free Expression of N-Term GFP SOX18, SOX7, SOX17 full length.

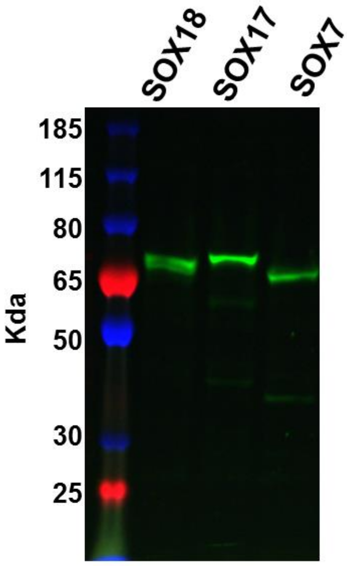

Figure S2: effect of the fluorophore on protein-protein interactions (PPIs).

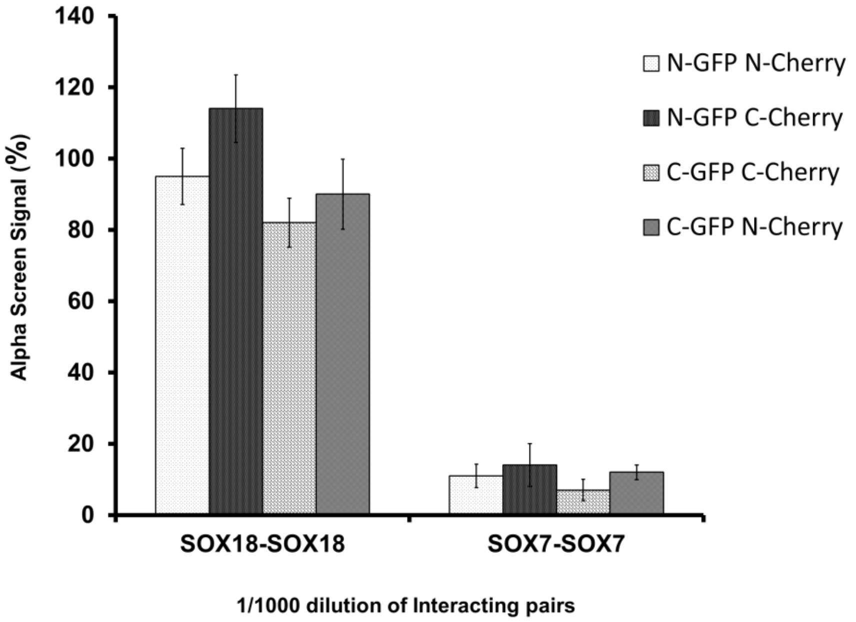

Figure S2: AlphaScreen signals obtained for different combination of tags for SOX18 and SOX7. All combinations showed that SOX18 was able to homodimerize whereas SOX7 was not.

Figure S3: Construction of the mVENUS-based SOX18 split-fluorescent protein (split-FP) biosensor.

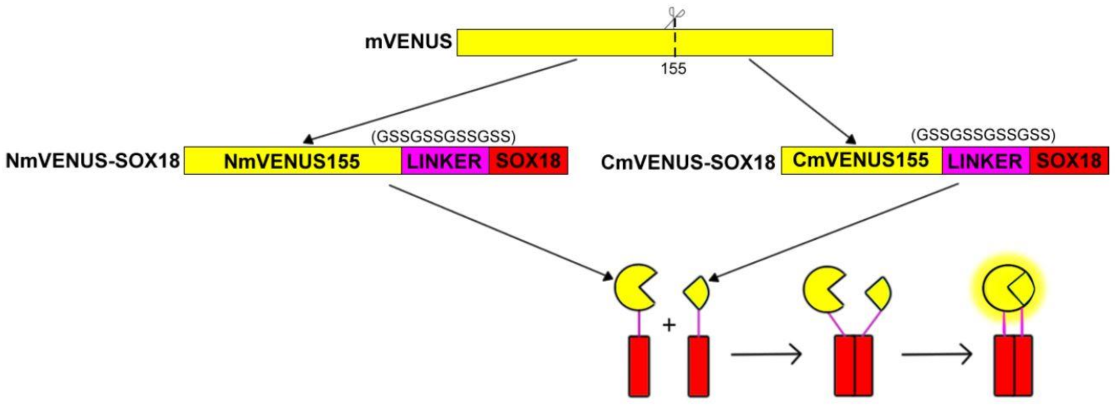

Figure S3: mVENUS was fragmented at amino acid 155 to give rise to an N-terminal fragment and a C-terminal fragment which were tagged onto the N-terminus of SOX18 via a flexible 3X GGGS linker to generate NmVENUS-SOX18S and CmVENUS-SOX18S biosensors respectively.

Figure S4: Typical AlphaScreen curves obtained for the SOX18 deletion mutants, lacking hydrophobic patches in the DIM domain.

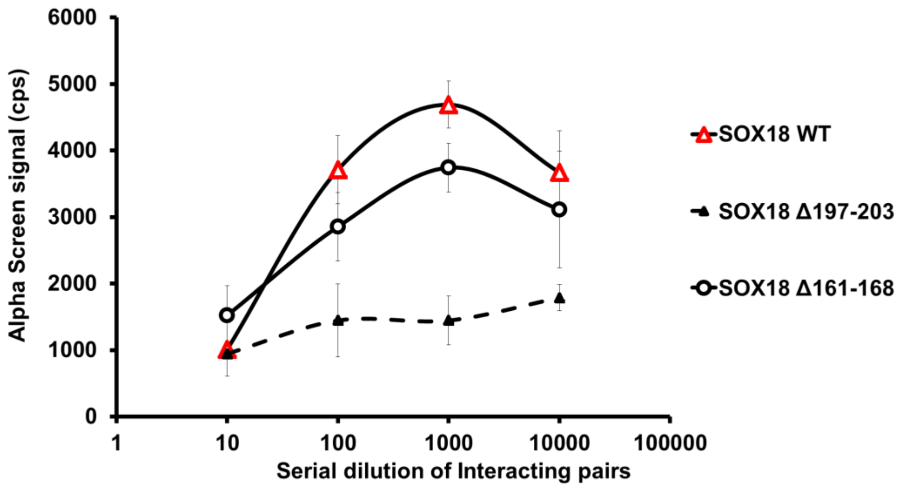

Figure S4: AlphaScreen curves were obtained for GFP- and Cherry- tagged pairs for SOX18 WT, SOX18Δ197-203 and SOX18Δ161-168. Both GFP-tagged SOX18 WT and SOX18Δ197-203 were able to bind SOX18-mCherry, whereas the lack of signal obtained for the SOX18/ SOX18Δ161-168 pair indicates a loss of the dimerization propensity.

Figure S5: Electrophoretic mobility shift assay for binding of SOX18-HMG and SOX9-HMG to IR5.

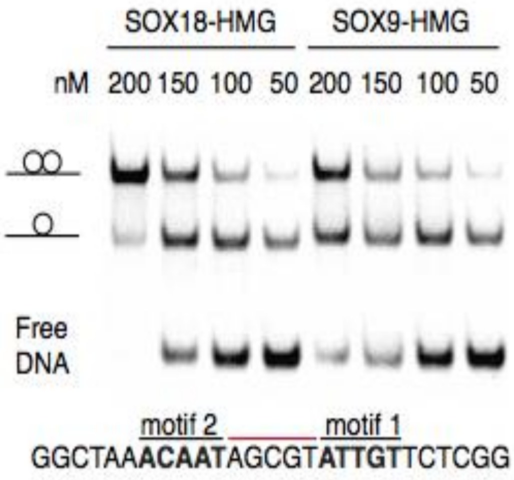

Figure S5: Purified, recombinant SOX18 and SOX9 HMG box constructs were tested for binding to oligonucleotides harbouring the palindromic SOX18 binding motif identified in Figure 4.B, separated by a 5 nucleotide spacer (IR5), revealing that two SOX18-HMG can bind. SOX 9 has a very similar HMG box and consensus-binding motif and therefore two SOX9 HMG can also bind to IR5.

Figure S6: Absence of effect of a single consensus SOX 18 binding motif on SOX18-RBPJ interaction.

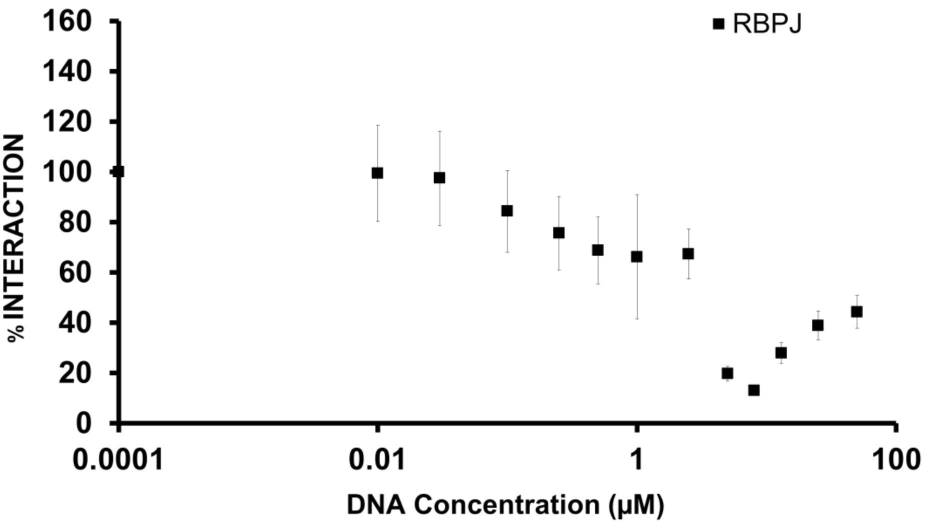

Figure S6: Maximum AlphaScreen signal for SOX18 WT – RBPJ as a function of concentration of single DNA consensus sequence.

Figure S7. GTEX analysis of the 964 SOX18 dimer-associated genes

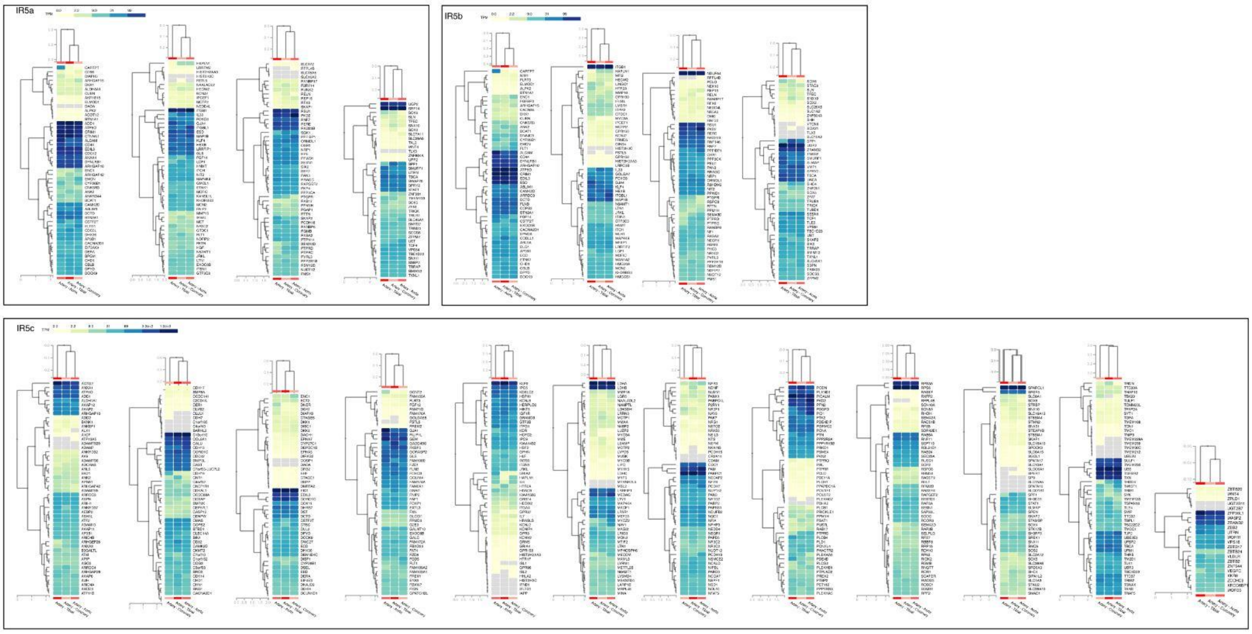

Figure S7. GTEX analysis for endothelial expression of the 964 genes associated to SOX18 ChIP-seq peaks containing a dimer motif (IR5a-c). Heat map shows the expression level of these genes in blood vessels. Blue corresponds to high expression levels; pale yellow is low expression levels.

Figure S8. Intersect between SOX18 ChIP-seq regions containing IR5 motif peaks and histone modifications or DNA hypersensitivity marks from HUVECs.

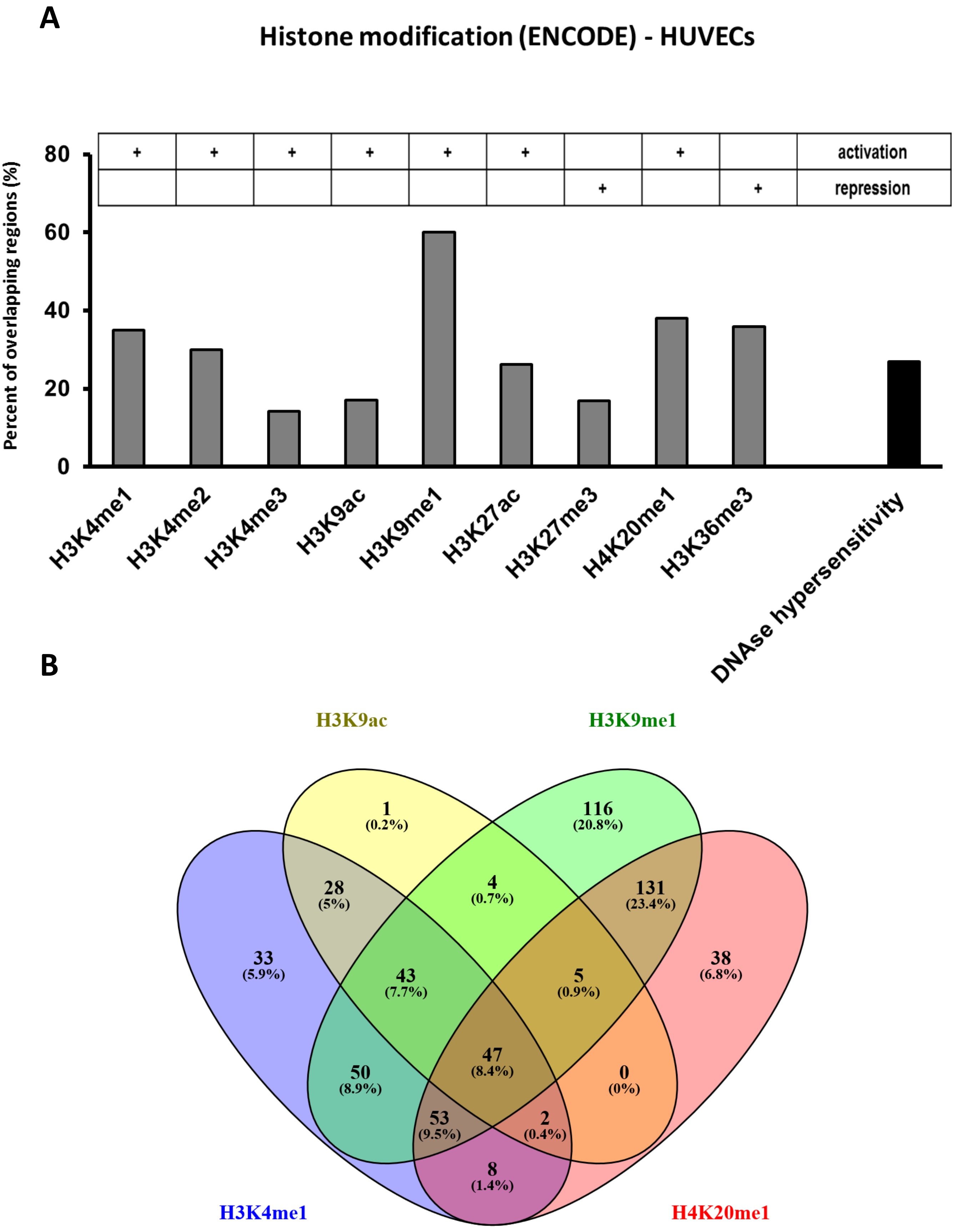

Figure S8. **(A)** Graph shows the intersection between SOX18 ChIP-seq peak locations and histone ChiPseq peaks coordinates, including H3K9ac (1),(2) and H3K4Me2 (3) using an overlap criterion of at least 50%. The overlap between SOX18 ChIP-seq peak locations and DNase hypersensitivity regions is also included. The intersection was performed using EpiExplorer online resource tool (4) based on ENCODE ChIP-seq data sets generated in HUVEC lines. **(B)** Venn diagram showing the intersection of different histone marks for active transcription that display at least 50% overlap with SOX ChIP-seq peaks which containing IR5a-c motifs, using Venny 2.1.0 (5).

Figure S9: Examples of genomic annotations in the promoter region of endomucin, an IR5 responsive gene.

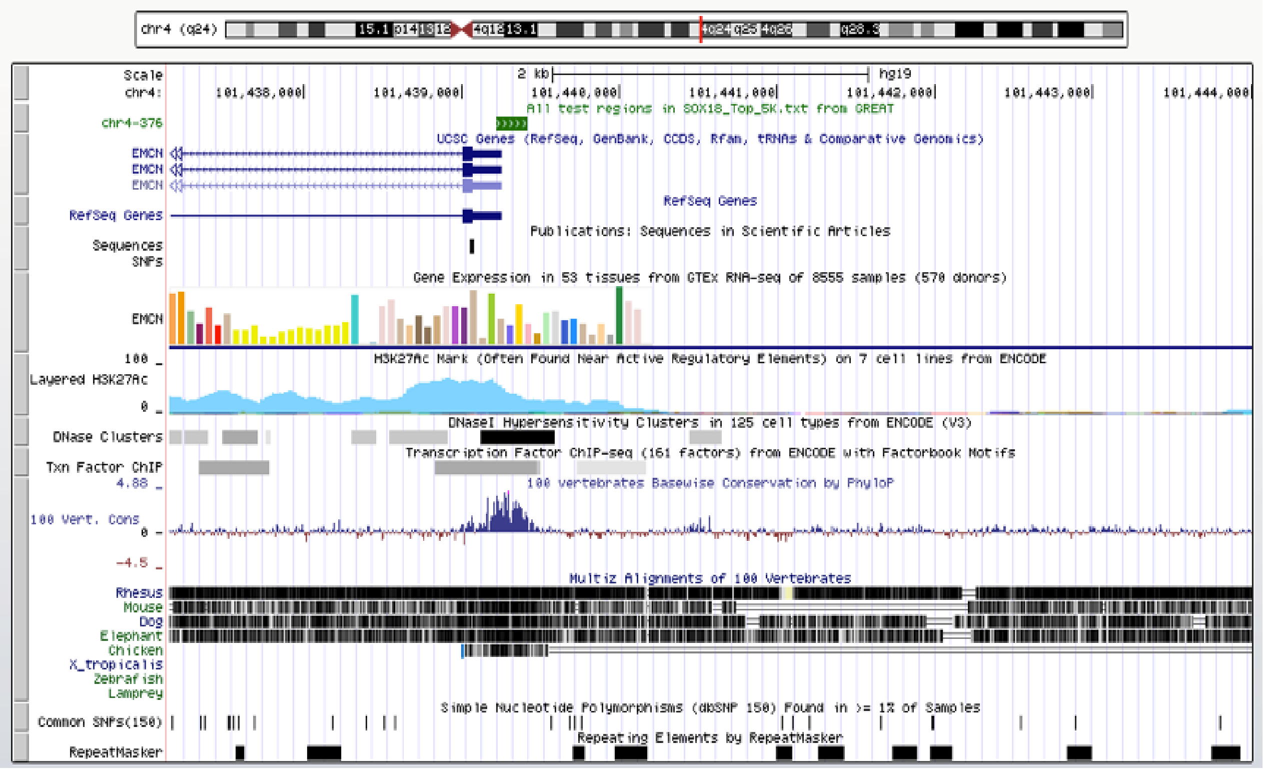

Figure S9: Example of a UCSC track for a SOX18 ChIP-seq peak (green bar) containing a dimer motif (IR5) assigned to the endomucin gene (dark blue bars). This ChIP-seq peak overlaps with active enhancer mark H3K27ac (blue shade) and a DNase hypersensitivity region (black bar). This putative regulatory region is highly conserved in birds and mammals (blue peaks and alignments, black stripes).

Figure S10: Supplementary figure to Figure 6.

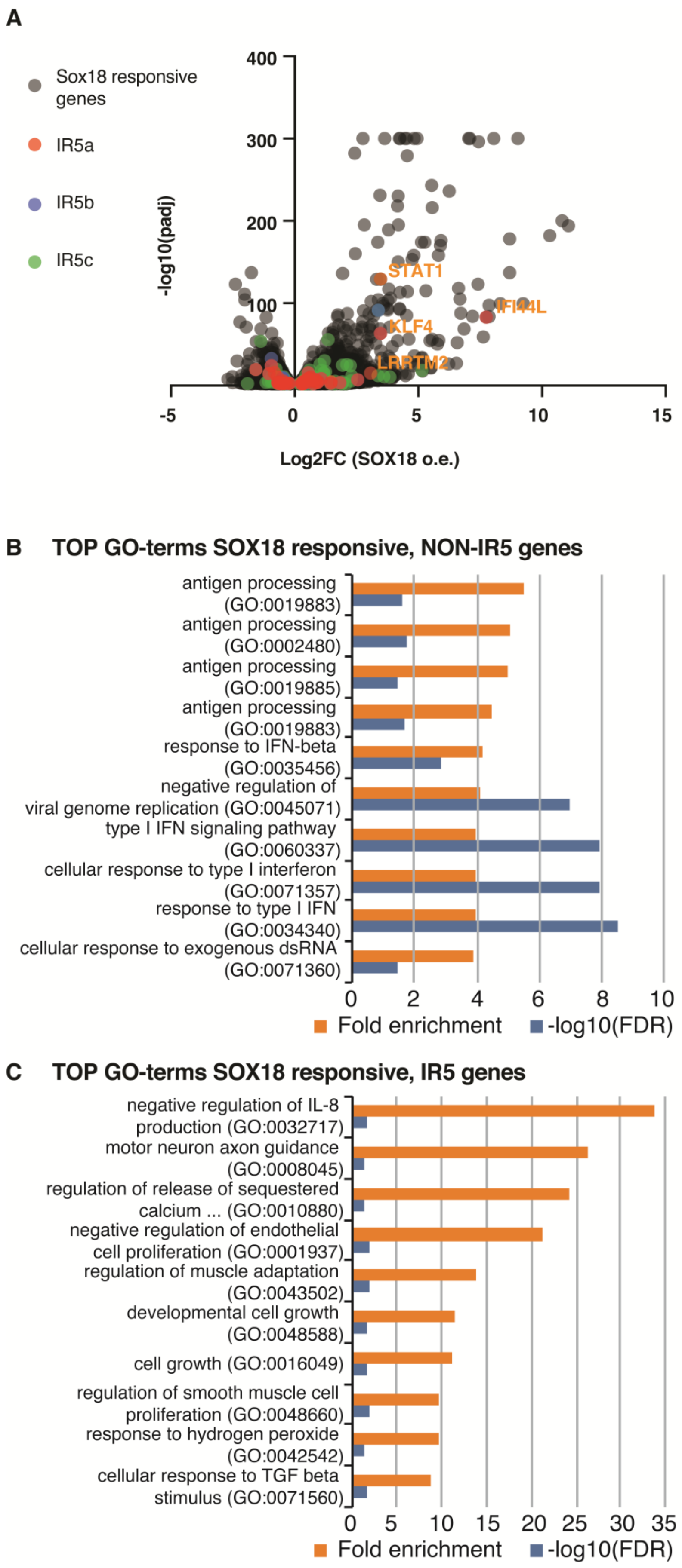

Figure S10: **(A)** Volcano plot (full version of Fig. 6A blow-up) showing all genes responsive to SOX18 o.e. in HUVECs. Genes with an IR5 motif have been highlighted in red (IR5a) blue (IR5b) and green (IR5c) and a selection of highly responsive genes has been annotated (e.g. *KLF4*). **(B, C)** Top 10 GO terms (PANTHER v13.1) of SOX18 responsive non-IR5 genes (A) and IR5 genes (B), revealing significant enrichment for biological processes related to the immune systems for non-IR5 genes and cell growth / proliferation (including endothelial) for IR5-genes (Fisher test, FDR < 0.05).

Table S1. List of primers used to create the different constructs of SOX18.

Names:	TM:	Sequences:
SOX18 Full length, N ter Forward	TM: 76.6	GGGG ACA AGT TTG TAC AAA AAA GCA GGC TTC atgcagagatccccgcccgg
SOX18 Nter Reverse	TM: 70.2	GGGG AC CAC TTT GTA CAA GAA AGC TGG GTT cctagattcgtctgcagcttgcc
SOX 18 HMG Reverse	TM: 74.6	GGGG AC CAC TTT GTA CAA GAA AGC TGG GTT ctgcttcttccgcctgggtctgtactt
SOX18 HMG Long Reverse	TM: 74.4	GGGG AC CAC TTT GTA CAA GAA AGC TGG GTT ggctcccaggggggcaactcgcg
SOX18 HMG box + (HMGBOX + TAD) Forward	TM: 77	GGGG ACA AGT TTG TAC AAA AAA GCA GGC TTC attcggcgccccatgaacgc
SOX18 HMG box Reverse	TM: 67.5	GGGG AC CAC TTT GTA CAA GAA AGC TGG GTT cttataattagggtgatccctcaaatgttg
SOX18 Full Length (HMG box + TAD) Reverse	TM: 75	GGGG AC CAC TTT GTA CAA GAA AGC TGG GTT cccggagatacaagcactgtagtagacggc
SOX18 TAD Forward	TM: 75.4	GGGG ACA AGT TTG TAC AAA AAA GCA GGC TTC tacagacccaggcggaagaagcagg

Table S1. The primers used to isolate the different domains of SOX18 are listed, along with their calculated melting temperature (TM) and sequences. All primers were obtained from IDT, as single stranded DNA fragments in form of lyophilized powder. This was then resuspended in Ultra-Pure distilled water (Invitrogen) to a final concentration of 10 µM before use.

Table S2. List of SOX18 ChIP-seq regions containing IR5 motif peaks overlapping with histone marks modifications.

Table S2. Excel sheet of coordinates of the overlapping regions.

Table S3: List of Sm4-responsive dimer genes

Table S3. Excel sheet of **Sm4** affected dimer genes (IR5a-c).

MOVIE 1*: *Dimerization process of the SOX18 homodimer observed via live imaging during zebrafish embryo development.

***MOVIE 1*:** Timelapse images of zebrafish embryos injected with the SOX18 homodimer split-fluorescent protein biosensor (NVENUS- /CVENUS-SOX18) imaged using confocal microscopy over a period of 10 hrs (left and middle). Timelapse is shown as both the fluorescent channel (514nm) alone (left), and as a composite of the fluorescent and brightfield channels (middle). Visualisation of the subcellular localization of the SOX18 homodimer in 4-5hpf zebrafish embryos reveal the association of SOX18 protein with chromatin of dividing cells (right). Examples of cells undergoing division where SOX18 appears to be strongly correlated with the chromatin are indicated by white arrows.

1. Consortium, E.P. (2007) Identification and analysis of functional elements in 1% of the human genome by the ENCODE pilot project. *Nature*, **447**, 799.
2. Heintzman, N.D., Stuart, R.K., Hon, G., Fu, Y., Ching, C.W., Hawkins, R.D., Barrera, L.O., Van Calcar, S., Qu, C. and Ching, K.A. (2007) Distinct and predictive chromatin signatures of transcriptional promoters and enhancers in the human genome. *Nature genetics*, **39**, 311.
3. Spicuglia, S. and Vanhille, L. (2012) Chromatin signatures of active enhancers. *Nucleus*, **3**, 126-131.
4. Halachev, K., Bast, H., Albrecht, F., Lengauer, T. and Bock, C. (2012) EpiExplorer: live exploration and global analysis of large epigenomic datasets. *Genome biology*, **13**, R96.
5. Oliveros, J.C. (2007) VENNY. An interactive tool for comparing lists with Venn Diagrams. *http://bioinfogp. cnb. csic. es/tools/venny/index. html*.
